# Spin Statistics for Triplet–Triplet Annihilation Upconversion: Exchange Coupling, Intermolecular Orientation, and Reverse Intersystem Crossing

**DOI:** 10.1021/jacsau.1c00322

**Published:** 2021-10-13

**Authors:** David G. Bossanyi, Yoichi Sasaki, Shuangqing Wang, Dimitri Chekulaev, Nobuo Kimizuka, Nobuhiro Yanai, Jenny Clark

**Affiliations:** † Department of Physics and Astronomy, The University of Sheffield, Hicks Building, Hounsfield Road, Sheffield S3 7RH, U.K.; ‡ Department of Chemistry and Biochemistry, Graduate School of Engineering, Center for Molecular Systems (CMS), Kyushu University, 744 Moto-oka, Nishi-ku, Fukuoka 819-0395, Japan; ¶ Department of Chemistry, The University of Sheffield, Dainton Building, Brook Hill, Sheffield S3 7HF, U.K.

**Keywords:** triplet−triplet annihilation, upconversion, spin statistics, rubrene, OLEDs, reverse intersystem crossing

## Abstract

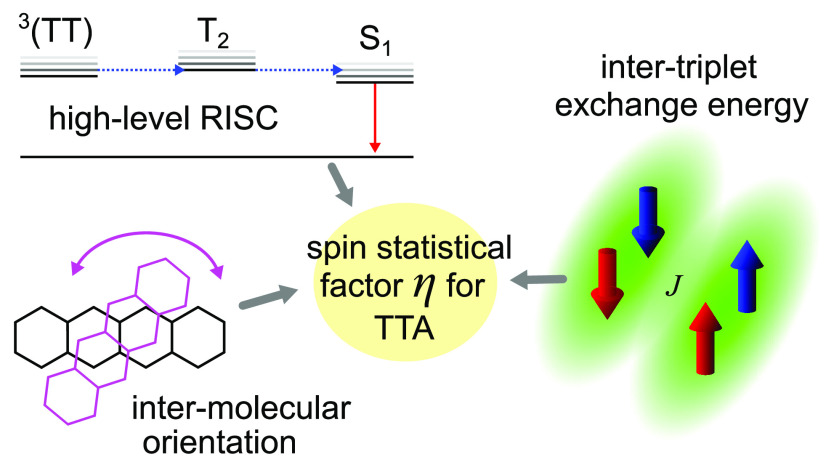

Triplet–triplet annihilation upconversion (TTA-UC) has great potential to significantly improve the light harvesting capabilities of photovoltaic cells and is also sought after for biomedical applications. Many factors combine to influence the overall efficiency of TTA-UC, the most fundamental of which is the spin statistical factor, η, that gives the probability that a bright singlet state is formed from a pair of annihilating triplet states. The value of η is also critical in determining the contribution of TTA to the overall efficiency of organic light-emitting diodes. Using solid rubrene as a model system, we reiterate why experimentally measured magnetic field effects prove that annihilating triplets first form weakly exchange-coupled triplet-pair states. This is contrary to conventional discussions of TTA-UC that implicitly assume strong exchange coupling, and we show that it has profound implications for the spin statistical factor η. For example, variations in intermolecular orientation tune η from  to  through spin mixing of the triplet-pair wave functions. Because the fate of spin-1 triplet-pair states is particularly crucial in determining η, we investigate it in rubrene using pump–push–probe spectroscopy and find additional evidence for the recently reported high-level reverse intersystem crossing channel. We incorporate all of these factors into an updated model framework with which to understand the spin statistics of TTA-UC and use it to rationalize the differences in reported values of η among different common annihilator systems. We suggest that harnessing high-level reverse intersystem crossing channels in new annihilator molecules may be a highly promising strategy to exceed any spin statistical limit.

## Introduction

Bright, emissive singlet excitons can be created from the fusion of two dark triplet excitons through the photophysical process of triplet–triplet annihilation (TTA).^1^ Efficient TTA is highly desirable for improving the performance of organic light emitting diodes (OLEDs)^2,3^ and solar photovoltaics^4−9^ as well as for biomedical applications,^10,11^ including targeted drug delivery^12^ and optogenetics.^13^ Furthermore, the interactions between triplet excitons that govern the TTA process are of fundamental interest to a variety of research areas such as the condensed phases of ground state triplet molecules,^14−17^ the physics of interacting bosons,^18^ quantum entanglement,^19,20^ and quantum information and computing based on organic molecules.^21,22^

The probability that a pair of annihilating spin-1 triplet excitons results in a spin-0 singlet exciton is given by the spin statistical factor, η, with 0 ≤ η ≤ 1. For OLEDs and TTA-mediated photon upconverters, materials systems with a high value of η would result in very efficient device performance.^2,3,7,8,23,24^ However, despite its fundamental importance, the triplet–triplet interactions that govern the value of η are not, in general, fully understood or appreciated. As a result, several potential strategies for designing materials with a high value of η have been largely overlooked to date.

The spin statistical factor of triplet–triplet annihilation, η, is almost always discussed in terms of nine pure-spin triplet-pair encounter complexes: one spin-0 singlet, three spin-1 triplets, and five spin-2 quintets.^24−29^ At first glance, this might suggest that ; however, measurements of triplet–triplet annihilation upconversion (TTA-UC) efficiencies greatly exceeding this limit demonstrate that this is not the case^25,30^ (in TTA-UC, annihilating triplets are first sensitized on acceptor molecules by energy transfer from photoexcited donor species^7,8,23,24^). As discussed further below, the quintet complexes readily dissociate again into individual triplets because molecular quintet states are energetically inaccessible in relevant molecules.^31^ The triplet complexes, on the other hand, can undergo internal conversion to nearby triplet states, leading to the loss of one triplet of the pair.^26,27^ If such internal conversion is efficient, this description yields .

These conventional discussions of spin statistics overlook many of the subtleties of triplet–triplet interactions, studied initially by Merrifield and coworkers 50 years ago^32^ and further developed by others since.^33,34^ Such interactions have been investigated in great depth more recently through research into the reverse process to triplet–triplet annihilation, singlet fission,^35,36^ whereby pairs of triplet excitons are produced from singlets via the same intermediate triplet-pair states.^37^ Here, we aim to bridge the apparent divide between the singlet fission and TTA-UC descriptions by demonstrating the profound effect of triplet-pair character, in particular the strength of intertriplet exchange coupling, on the spin statistical factor. Inspired by recent reports of high-level reverse intersystem crossing from T_2_ to S_1_,^38^ which could allow the loss associated with the formation of triplet complexes to be bypassed,^39^ we also investigate internal conversion rate constants and the fate of higher-lying triplet states and their impacts on the spin statistical factor.

We therefore begin by providing an overview of the spin physics of triplet-pair states in the context of TTA-UC. Next, we investigate the triplet-pair character, energy levels, internal conversion rate constants and reverse intersystem crossing in rubrene, the most common acceptor molecule for near-infrared-to-visible TTA-UC.^8,29^ Based on these experimental results, we present an updated model for the spin statistics of upconversion that includes the effects of intertriplet exchange coupling and orientation, as well as internal conversion rate constants, energy levels and reverse intersystem crossing. We find that variations in exchange energy and orientation can tune the spin statistical factor η within the range , but that careful optimization of the S_1_, T_2_, and T_1_ energy levels may allow η to reach unity, thereby bypassing such considerations.

## Theoretical Background

Recent reviews have discussed the current understanding of the spin physics of triplet-pair states in great depth.^35,36^ Here, we review the important points and relate them to the spin statistical factor η.

Individual triplet states are governed by a spin Hamiltonian comprising (in the absence of spin–orbit coupling and other perturbations), a Zeeman term describing the effect of external magnetic fields **B**, and an intratriplet dipole–dipole coupling term, parametrized by the so-called zero-field splitting parameters *D* and *E*:
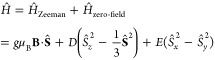
1where **Ŝ** is the 2-electron spin operator. In the **B** = 0 limit, the three triplet eigenstates are given by
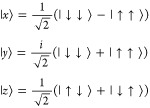
2where the arrows indicate the individual electron spin states. Because we use rubrene as our model system, we define our coordinate system such that *x* is parallel to the long molecular axis, *y* is parallel to the short axis, and *z* is perpendicular to the tetracene backbone plane.^40^

The spin Hamiltonian for a pair of interacting triplet states, labeled *A* and *B*, can be written as
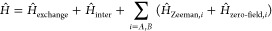
3In addition to the 2-electron spin Hamiltonians (eq 1) for individual triplets on molecules *A* and *B*, there are two additional intertriplet terms that couple their spins together. First, there is an intertriplet exchange interaction of strength *J* which requires wave function overlap between the two triplets in the pair. Second is an intertriplet spin-dipolar coupling term, which is a longer range, through-space interaction. The total spin Hamiltonian becomes^36^
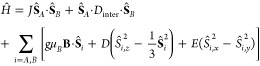
4The spin-dipolar term can be formulated in various ways. Because the intertriplet coupling strength, which we label *X*, is thought to be on the order of 10 neV,^41,42^ much less than the intratriplet dipolar coupling (*D* ∼ 10 μeV, *E* < *D*), the exact form is unimportant.^40^ For simplicity, we take **Ŝ**_*A*_·*D*_inter_·**Ŝ**_*B*_ ≈ *X***Ŝ**_*A*_·**Ŝ**_*B*_.^43^

A convenient basis set for diagonalizing *Ĥ* and obtaining the triplet-pair spin wave functions |ψ_*l*_⟩ comprises the nine product pair states |*xx*⟩, |*xy*⟩, ..., |*zz*⟩, where we have dropped the *A*, *B* subscripts for clarity. We note that because the *xyz* coordinate systems of molecules *A* and *B* do not in general coincide, a rotation operation must be applied to *Ĥ*_zero-field,*B*_.^40^ As a result, |ψ_*l*_⟩ carry a dependence on the relative orientation of the two molecules which, as we demonstrate below, has important implications for the spin statistical factor η. From here on in, we define “parallel” molecules as a pair for which molecule A can be mapped onto molecule *B* by means of a translation operation only; in other words their molecular coordinate systems coincide.

Recent research has shown the importance of distinguishing between strongly (*J* ≫ *D*) and weakly (*J* ≪ *D*) exchange-coupled triplet-pair states.^44−47^ In the limit of strong exchange coupling, the eigenstates |ψ_*l*_⟩ of *Ĥ* coincide with the nine lowest energy eigenstates of the four-electron spin operator (**Ŝ**_*A*_ + **Ŝ**_*B*_)^2^. They are therefore pure spin states (spin is a good quantum number) and comprise one spin-0 singlet ^1^(TT), three spin-1 triplets ^3^(TT) and five spin-2 quintets ^5^(TT). In the zero-field basis, the spin wave functions can be written as^48^
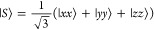
5
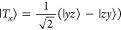
6
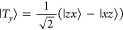
7
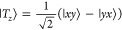
8
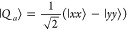
9
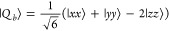
10
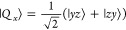
11
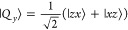
12
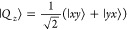
13and the triplet and quintet states are separated in energy from the singlet by *J* and 3*J*, respectively.^46,49^ These are the nine triplet-pair intermediates that are usually considered when evaluating the spin statistics of TTA.^24−29^ Thus, to date, there has been an implicit assumption within the TTA-UC community that the encounter complexes formed through TTA are strongly exchange-coupled.

One notable exception is the formulation provided in 1975 by Atkins and Evans.^34^ They extended the original Johnson-Merrifield model^32^ to explicitly include the effects of intertriplet exchange coupling and rotational dynamics of the molecules. Their analysis provides a useful framework for understanding magnetic field effects arising in solution phase TTA.^50,51^

One of the main conclusions of Atkins and Evans is that quintet-singlet crossing is inefficient for large values of *J*(34) which is unsurprising given the energy separation between them of 3*J*.^46,49^ When *J* ≪ *D*, however, the eigenstates of *Ĥ* become degenerate and possess very different spin character.^49^ In the case of parallel molecules (related by a translation operation only), there are three pure triplets |*T*_*x*,*y*,*z*_⟩ and three pure quintets |*Q*_*x*,*y*,*z*_⟩. The remaining three, |*xx*⟩, |*yy*⟩, and |*zz*⟩, can be written as mixtures of |*S*⟩, |*Q*_*a*_⟩ and |*Q*_*b*_⟩. In other words, we can no longer consider pure spin states. For nonparallel molecules (translation plus rotation), additional singlet–triplet and quintet-triplet mixing occurs and all of the eigenstates obtain mixed spin character.^46^ We can quantify the character of the eigenstates by calculating their overlap with the appropriate pure spin states. For example the singlet character is given by^32,52^
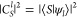
14

Analogously, we can define the triplet character as^53^
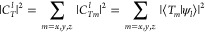
15and the quintet character as
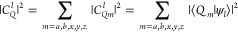
16

To understand the influence of triplet-pair character on the spin statistics of TTA-UC, we can construct a kinetic model based on the Johnson–Merrifield framework.^32,52^ We note that while related analyses have been reported by Mezyk et al. in 2009^53^ and more recently by Schmidt and Castellano,^54^ the effect of triplet-pair character on spin statistics was not explored in either work. Even in the work of Atkins and Evans,^34^ in which the intertriplet exchange interaction was explicitly included, the spin statistical factor (written by them as λ_*T*_) was incorporated only as a parameter and no expression for it was ever given.

**Figure 1:**
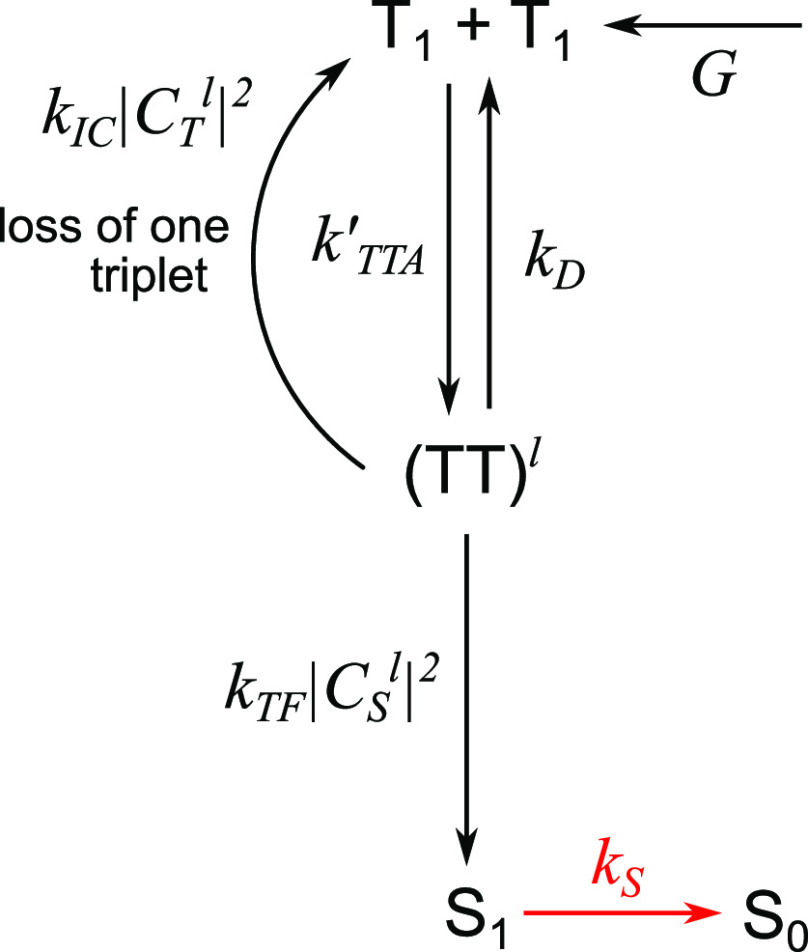
The simplest kinetic model of TTA-UC. A schematic diagram of the simplest kinetic model for TTA-UC that considers triplet-pair spin character in a general way. The processes and rate constants are described in the text. This is referred to as model 1.

The simplest possible model is illustrated in Figure 1. Triplet states generated at rate *G* can annihilate to form triplet-pair states (TT)^*l*^, whose spin wave functions |ψ_*l*_⟩ are determined by eq 4. We choose to consider an annihilation process that depends linearly rather than quadratically on the triplet population. This results in a linear set of rate equations with a simple analytical solution under steady-state conditions. TTA is therefore described by an effective annihilation rate constant *k*_TTA_^′^. The final expression for the spin statistical factor (eq 21, below) is identical to that obtained if we instead use a bimolecular, quadratic TTA process.

The triplet-pair states formed can either dissociate back into independent triplets with rate constant *k*_*D*_ or form a singlet state with rate constant *k*_TF_, modulated by the singlet character |*C*_S_^*l*^|^2^. The singlets decay radiatively with rate constant *k*_S_. We also include an internal conversion channel, with overall rate constant *k*_IC_, that results in the loss of one triplet; participation in this channel requires nonzero triplet character so the rate constant is modulated by |*C*_T_^*l*^|^2^. Quintet triplet-pairs are approximately equal in energy to S_1_ only when two chromophores are involved. Coalescence from a quintet triplet-pair to a single-chromophore molecular quintet state is energetically infeasible^31^ (the corollary for singlets and triplets is opposite). The only fate of quintets is therefore to simply break apart again into independent triplets^26^ and so no process in the model depends explicitly on the quintet character of the triplet-pair states. For interested readers, Supporting Information Section 5 explains this assumption further in the context of recent time-resolved electron paramagnetic resonance studies^55−58^ that claim direct interconversion between ^1^(TT) and ^5^(TT).

The rate equations describing the above processes can be written as follows:
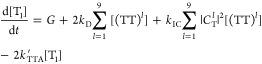
17
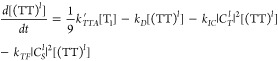
18
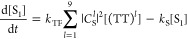
19Because the photoluminescence quantum yield (PLQY) is unity in this model and no other losses are present besides spin statistical effects, the spin statistical factor η can be evaluated analytically by solving the equations under steady-state conditions. We obtain
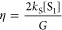
20
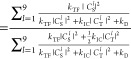
21Equation 21 is identical to the expression previously arrived at by Schmidt and Castellano,^54^ though it was not written out explicitly in their work. At the time, however, the distinction between weak and strong exchange coupling within triplet-pair states was not so well understood, and the true implications were not fully grasped.

We can evaluate eq 21 for the limits of strongly and weakly exchange-coupled triplet-pairs discussed above. We find
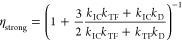
22
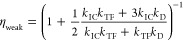
23Assuming that the dissociation of triplet-pair states is considerably slower than fusion or internal conversion (*k*_D_ ≪ *k*_TF_, *k*_*IC*_), we obtain, as expected,^27,54^ in the limit of strong exchange coupling. Interestingly, however, the spin statistical factor rises to  for weakly exchange-coupled triplet pair states. In both cases, η = 1 if *k*_IC_ = 0.

**Figure 2:**
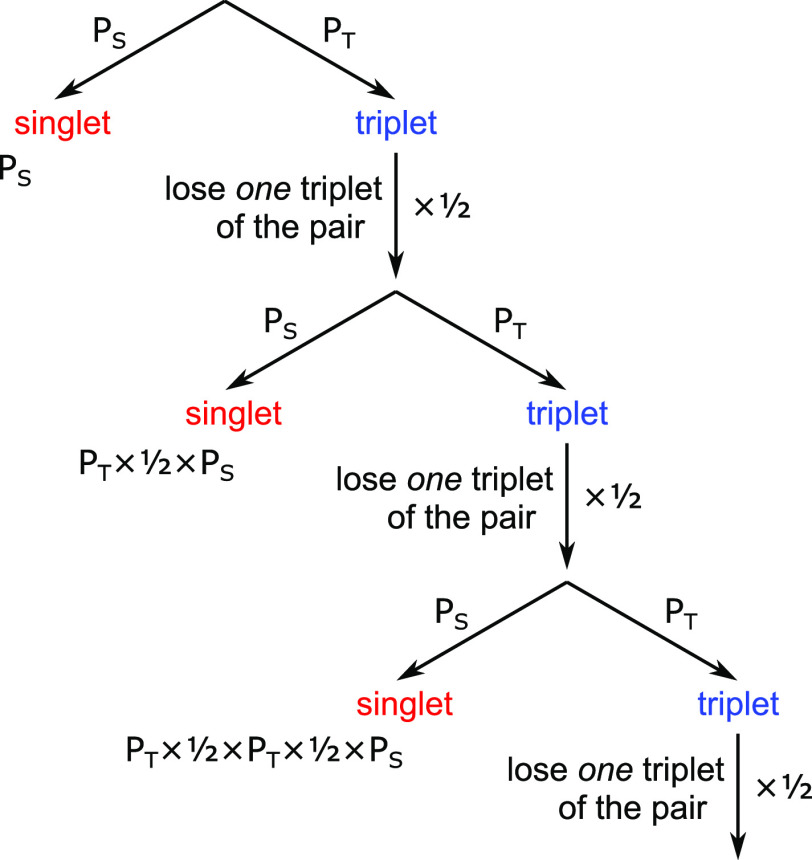
Probability tree for TTA spin statistics. The spin statistical factor can be evaluated using a probability diagram when the triplet character is contained exclusively in pure spin-1 states (there is no triplet-quintet or triplet-singlet mixing, as is the case for molecules oriented parallel). *P*_S_ and *P*_T_ are the respective probabilities of forming a triplet-pair state with singlet or triplet character.

We can understand these limits more intuitively by considering the probability tree associated with triplet-pair formation events (Figure 2). Only triplet-pair states with singlet or triplet character are “active” in TTA-UC and we let their probabilities of formation be *P*_S_ and *P*_T_ respectively. The spin statistical factor is then given by a geometric progression:
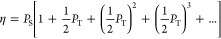
24
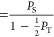
25

In the case of strong exchange coupling, the relevant triplet-pair states comprise one pure singlet and three pure triplets, giving  and , and hence . For weakly exchange coupled triplet-pair states (on parallel molecules), we again have three pure triplets. The singlet character is spread across three singlet-quintet mixtures. The quintet component does not affect the fate of these mixed spin states, and so we have  and therefore .

Equations 21–23 allow us to identify the key factors expected to affect the spin statistics of TTA-UC. First, the intertriplet exchange energy *J* determines the character of the triplet-pair spin wave functions. If *J* is negligible compared to other terms in the spin Hamiltonian (eq 4), the finer details of the intratriplet spin dipolar interactions, including intermolecular orientation, also play a role. Second, the rate constants of internal conversion from ^3^(TT) to individual triplet states T_N_, and the subsequent fate of T_N_, have a profound effect. If the internal conversion is slow in comparison to triplet-pair fusion and separation, or if high-level reverse intersystem crossing^38,39,59,60^ (HL-RISC) channels ^3^(TT) states to S_1_ via T_2_, the spin statistical factor can approach unity.^39^ In the following, we investigate these factors in turn in the context of rubrene, the most common acceptor molecule for near-infrared-to-visible TTA-UC.

## Results

**Figure 3:**
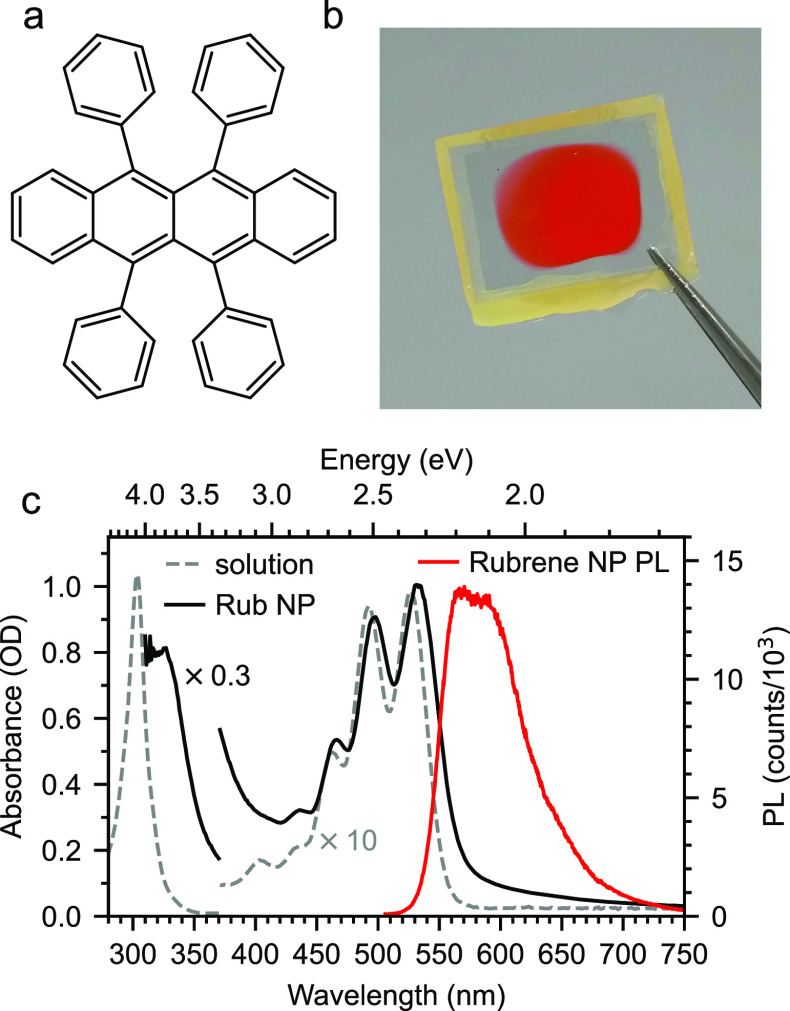
Rubrene nanoparticle films. (a) Molecular structure of rubrene. (b) Photograph showing a film of rubrene nanoparticles dispersed in an oxygen-blocking PVA matrix and cast onto a glass substrate. The sample is covered with a thin glass slip and sealed with epoxy resin. (c) Absorption and emission spectra of rubrene nanoparticle films alongside the absorption spectrum of rubrene dissolved in toluene (10^–4^ M).

Figure 3a shows the molecular structure of rubrene. In crystalline rubrene, triplets are formed via singlet fission on the picosecond time scale,^61−63^ allowing their fusion behavior to be studied without the presence of sensitizer species.^64^ We perform the majority of our experiments on rubrene nanoparticles (NPs) dispersed in a poly(vinyl alcohol) (PVA) matrix (Figure 3b). Nanoparticles prepared in this way (see Experimental Section) have an average diameter of 220 nm and show no sharp peaks in their X-ray diffraction pattern.^65^ These nanoparticle films are the basis of recently reported solid-state TTA-UC systems.^65,66^

In Figure 3c we present the absorption and emission spectra of the rubrene NPs alongside the absorption spectrum of rubrene monomers in toluene. From these spectra, we confirm the S_1_ energy level at between 2.32 and 2.23 eV based on the absorption and emission maxima, respectively. A small peak at 400 nm (3.1 eV) is clearly visible in the solution absorption spectrum which does not appear to follow the vibronic progression of the S_1_ state. We suggest that this may be a signature of S_2_ and that the strong absorption at around 300 nm (4.13 eV) corresponds to a higher-lying S_0_ → S_N_ transition.

### Triplet-Pair Character

Equations 21−23 demonstrate that the spin Hamiltonian of eq 4, in particular the intertriplet exchange coupling *J*, has a profound effect on the spin statistical factor η. To probe the intertriplet interactions in our rubrene NPs, we measured the effects of magnetic fields on the delayed fluorescence during bimolecular triplet–triplet annihilation.

**Figure 4:**
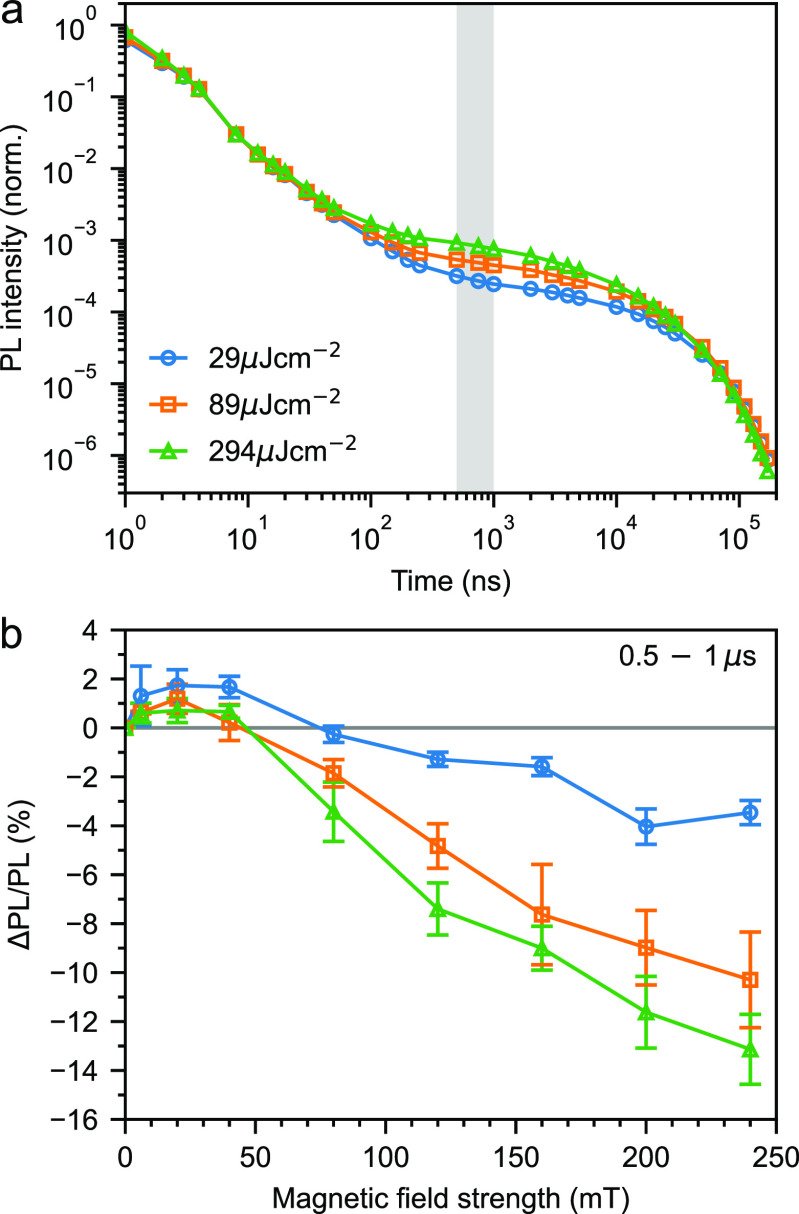
Triplet–triplet annihilation and magnetic field effects. (a) Time-resolved PL of a rubrene nanoparticle film at three different excitation intensities. The decays have been normalized at 8 ns. (b) MFEs on fluorescence gated from 0.5 to 1 μs at the same three excitation intensities. Error bars reflect the variation between sweeping up and down in magnetic field and arise from slight photobleaching and small fluctuations in laser power.

Figure 4a shows the time-resolved photoluminescence (PL) of a rubrene NP film at three different excitation intensities. Between 100 ns and 10 μs, we find that greater excitation density leads to a relative increase in measured PL. These dynamics are characteristic of bimolecular triplet–triplet annihilation that, via triplet-pair intermediates, repopulates the S_1_ state.^37^

To investigate the character of the triplet-pair states that are the initial product of bimolecular TTA, in Figure 4b we plot the change in PL intensity 0.5 to 1 μs after excitation as a function of applied magnetic field, at the same three excitation intensities as Figure 4a. We observe a small increase in the PL for fields <50 mT followed by a decrease at higher fields. The overall magnitude of the effect increases with excitation intensity, demonstrating that the triplet-pairs responsible are products of bimolecular TTA.

Magnetic field effects (MFEs) such as those presented in Figure 4b are well-known to be characteristic of triplet–triplet annihilation and were first explained by Johnson and Merrifield 50 years ago.^32,67^ Their model for the spin physics of singlet fission and triplet–triplet annihilation is based on the spin Hamiltonian (eq 4) *but with no exchange term*, i.e. *J* = 0. Thus, Johnson and Merrifield’s rather vaguely defined “TT” states are implicitly weakly exchange-coupled, though such terminology was not used at the time. As implied in the later work of Atkins and Evans,^34^ MFEs measured under fields of a few tens of mT are therefore signatures of weakly exchange-coupled triplet-pair states.^46^ This can be readily understood by examining the spin Hamiltonian of eq 4.

The zero-field splitting parameter *D* is typically around 10 μeV. For example, it is 6.45 μeV in tetracene^68^ and is thought to be similar for rubrene.^40^ The Zeeman term thus has a similar magnitude to the zero-field term when *gμ*_B_*B* ∼ *D*, i.e. *B* ∼ 50 mT. In the absence of other terms in the spin Hamiltonian of similar or greater magnitude, the competition between the Zeeman and zero-field terms at such fields leads to variations in the eigenstates |ψ_*l*_⟩ with magnetic field and hence to variations in the singlet character |*C*_S_^*l*^|^2^ (eq 14) of the triplet-pair states.^32,52^ For example we have seen that when *B* = 0, three of the eigenstates (|*xx*⟩, |*yy*⟩, and |*zz*⟩) have singlet character. If *gμ*_B_*B* ≫ *D*, this falls to two,^32^ giving rise to the characteristic reduction in measured PL during triplet–triplet annihilation. If, however, as is implicitly assumed in discussions of spin statistics for TTA-UC, the triplet-pairs formed are all strongly exchange-coupled (*J* ≫ *D*), we would not see any significant MFE until *gμ*_B_*B* ∼ *J*, because the zero-field term now acts only as a tiny perturbation. This requires much higher field strengths and gives rise to very different types of MFE.^46−48^ In acene materials, high-field MFEs have been reported in only one material, TIPS-tetracene^47^ and the effect was observed only at 1.4 K.

MFEs corresponding to weakly exchange-coupled triplet-pairs, similar to ours in Figure 4b, have been measured during TTA-UC both in the solid state^53^ and in solution.^50,51,69,70^ Observations of these MFEs cannot prove that all TTA events exclusively produce triplet-pair states that are initially weakly exchange-coupled because strongly exchange-coupled triplet-pairs do not contribute to the MFE at low (tens to hundreds of mT) magnetic field strengths. Nevertheless, the idea that triplet-pair states formed through bimolecular TTA are weakly exchange-coupled is supported by our previous work^37^ and it is demonstrably true for at least a proportion of TTA events. Below, we therefore explore the implications of this for the spin statistics of TTA-UC. First, however, we investigate the other key factors that may impact the spin statistical factor: internal conversion, energy levels and reverse-intersystem crossing.

### Energy Levels and Internal Conversion

To estimate the rate constants of internal conversion from ^3^(TT) to T_N_, we must first determine the triplet energy levels. The energy of T_1_ is well-known to be 1.14 eV for rubrene.^71−73^ We can therefore take the energy of ^3^(TT) to be 2.28 eV in the absence of large intertriplet binding. Reported values for the rubrene T_2_ energy vary significantly.^74−77^ For a precise determination of the higher lying triplet energies, we turn to transient absorption (TA) spectroscopy.

**Figure 5:**
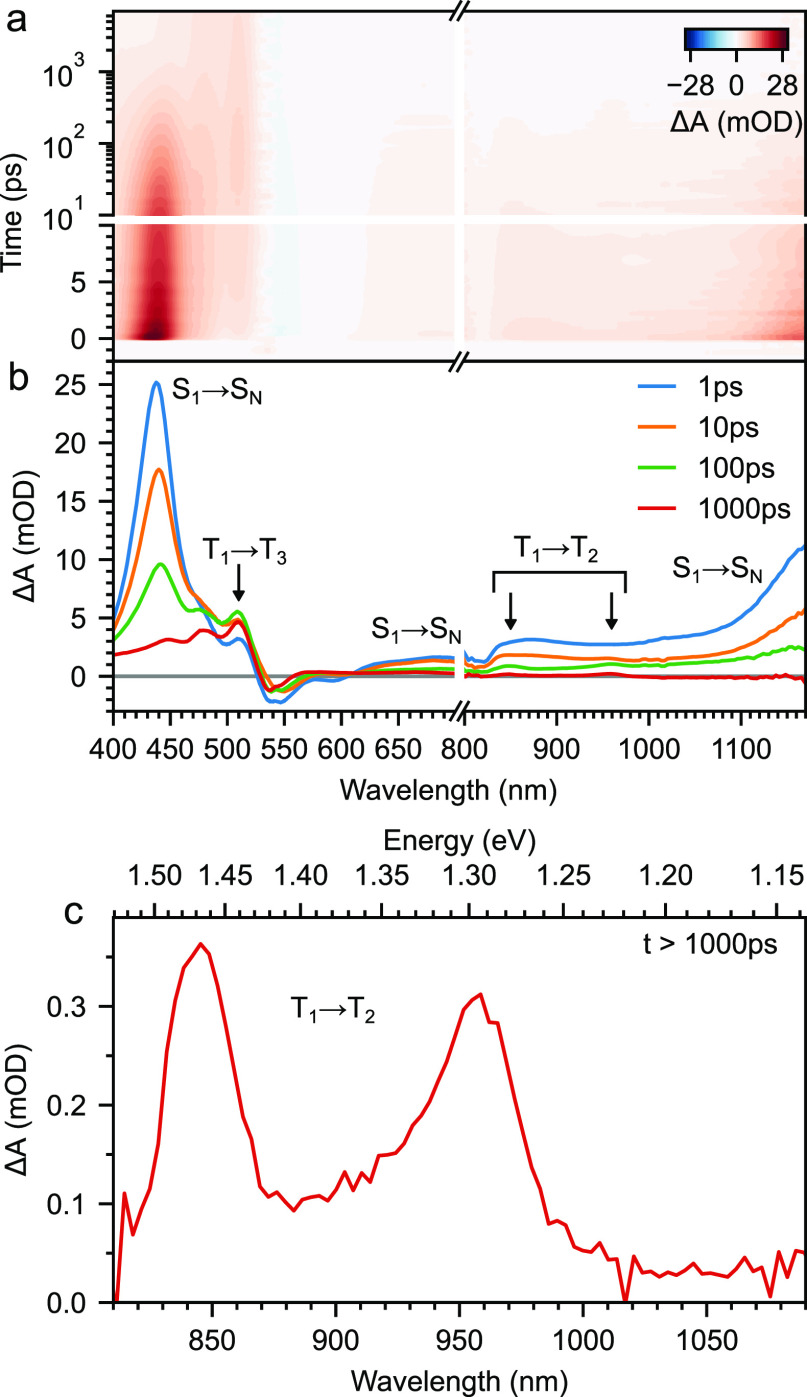
Transient absorption spectroscopy of rubrene nanoparticle films. (a) False-color map showing transient absorption measurements of rubrene NP films pumped at 532 nm with an excitation intensity of 40 μJ cm^–3^. (b) Transient absorption spectra spanning the visible and near-infrared reveal singlet fission dynamics. Singlet PIA features at 440, 680, and 1170 nm decay, accompanied by a rise in triplet PIA bands at 510, 850, and 960 nm. The latter two peaks, highlighted in (c), correspond to the 0-0 and 0-1 bands of the T_1_ → T_2_ transition. Given the T_1_ energy of 1.14 eV, we calculate the T_2_ and T_3_ energy levels to be 2.43 and 3.57 eV, respectively.

Figure 5 shows transient absorption spectra of a rubrene NP film pumped at 532 nm. We find the characteristic signatures of singlet fission in rubrene: the singlet photoinduced absorption (PIA) at 440 nm decays rapidly, accompanied by a rise in the triplet PIA at 510 nm.^61^ Broad PIA features at around 680 and 1170 nm decay with similar dynamics to the 440 nm band (Figure S1) and we therefore assign them to S_1_ → S_N_ transitions, as reported previously.^63,73^ Finally, we observe two PIA peaks in the near-infrared at 960 and 850 nm (Figure 5c) whose dynamics match those of the well-known triplet PIA at 510 nm (Figure S1). Similar peaks have previously been assigned to triplet states in rubrene.^73^ Broad PIA features in the same spectral region have been explicitly assigned to T_1_ → T_2_ transitions in rubrene,^63^ in agreement with calculations.^78^ The two sharp peaks that we measure here are separated in energy by 0.17 eV, suggesting that they belong to a vibronic progression. We therefore assign them to the 0–0 and 0–1 vibronic peaks of the T_1_ → T_2_ transition, putting the T_2_ energy at 2.43 eV. The next triplet PIA is that at 510 nm, suggesting that T_3_ lies at 3.57 eV.

**Figure 6:**
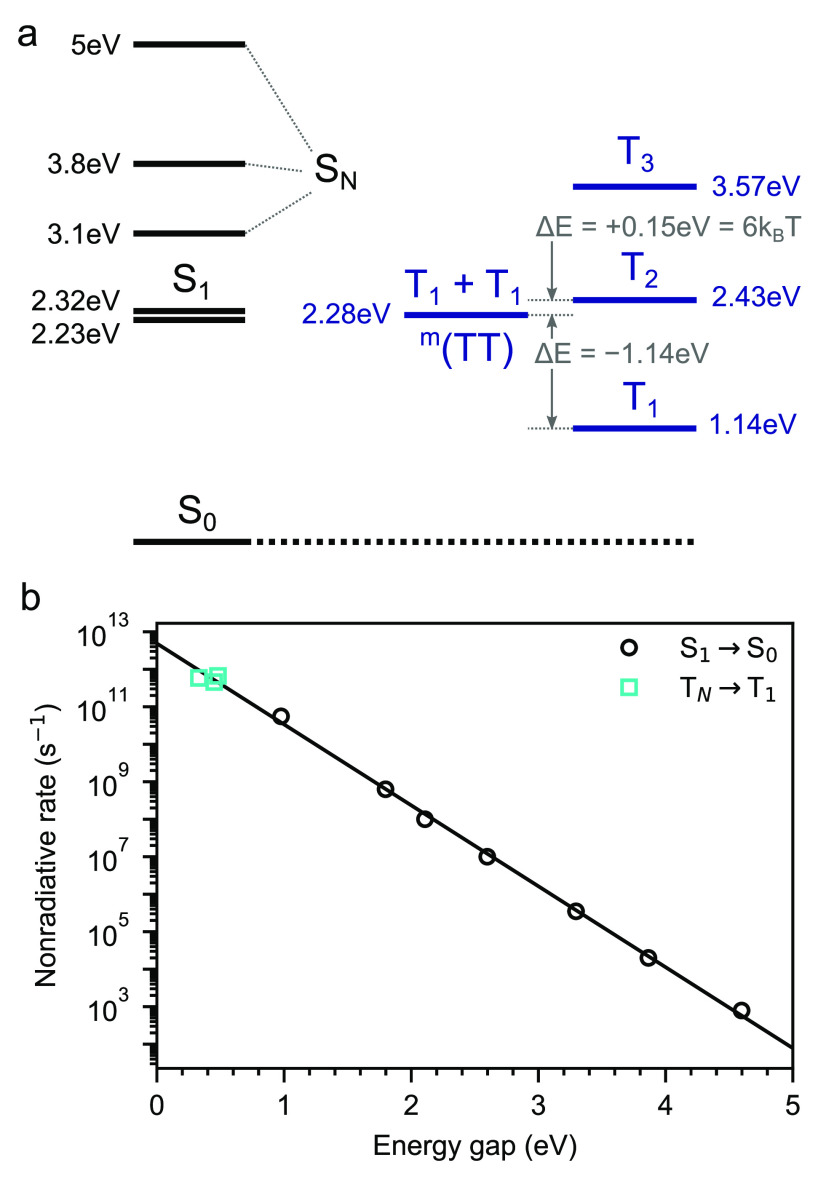
Energy levels of rubrene and internal conversion in acenes. (a) Energy level diagram for rubrene based on the transient absorption spectra in Figure 5. ^3^(TT) → T_1_ is exothermic by 1.14 eV and ^3^(TT) → T_2_ is endothermic by 150 meV = 6*k*_B_*T*. (b) S_1_ → S_0_ nonradiative rates plotted against optical gap for acenes based on data in refs.^79^ and.^80^ We find excellent agreement with the energy gap law. Measurements of triplet–triplet internal conversion rate constants in erythrosin B, rose bengal, and tetraphenylporphyrin^59^ follow the same gap law.

We use the photoinduced absorptions from Figure 5 to construct the energy level diagram of rubrene shown in Figure 6a. Of particular importance for the spin statistics of upconversion are the energy differences between 2T_1_ ≈ ^3^(TT), T_1_ and T_2_. ^3^(TT) → T_1_ is exothermic by 1.14 eV, while ^3^(TT) → T_2_ is endothermic by 150 meV = 6*k*_*B*_*T*. To date, only the relative energy levels have been considered in determining whether the ^3^(TT) → T_N_ loss channel is operational in TTA-UC.^28^ Here, we aim to go a step further by estimating the rate constants of the internal conversions.

In the absence of strong vibronic or nonadiabatic coupling, the rate constant of internal conversion in organic molecules obeys the energy gap law,^81^ which we write as
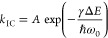
26where Δ*E* is the energy gap between the electronic states, *ℏω*_0_ is the highest available vibrational frequency that couples to the electronic states (taken to be the symmetric vinyl stretching mode at 0.17 eV)^82^ and γ and the prefactor *A* are material system dependent.

We begin by assuming that internal conversion in the triplet and singlet manifolds obeys the same energy gap law. For singlet internal conversions, we use the rate constants of the nonradiative S_1_ → S_0_ transition. These have been determined experimentally for the acene family from benzene through to hexacene^79^ and also for carbon nanotubes.^80^ Following ref (36)., we plot these internal conversion rate constants against their optical gaps in Figure 6b and find excellent correspondence with the energy gap law. This allows us to extract values of *A* = 4.9(13) × 10^12^ s^–1^ and γ = 0.845 ± 0.015 for molecules comprising fused aromatic rings. Experimental determinations of triplet–triplet internal conversions are much less common, though measurements do exist for erythrosin B, rose bengal, and tetraphenylporphyrin.^59^ Plotting these values on Figure 6b, we find good agreement with the energy gap law for singlet manifold internal conversions, providing some justification of our earlier assumption.

We use the values of *A* and γ extracted from Figure 6b in eq 26 to estimate the triplet internal conversion rate constants in rubrene. For the exothermic ^3^(TT) → T_1_ process, we find a rate constant of 1.7(5) ± 10^10^ s^–1^ or 60(20) ps. The endothermic route via T_2_ requires thermal activation but can then proceed with an energy gap of zero. Thus, the rate constant can be approximated by
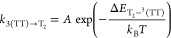
27which evaluates to 1.2(3) ± 10^10^ s^–1^ or 80(20) ps at room temperature. The internal conversion rate constants are therefore expected to be similar for transitions to T_1_ and T_2_ despite the endothermic nature of the latter. This is highly significant: it has been recently reported that HL-RISC from T_2_ to S_1_ can occur in rubrene,^38^ potentially providing a pathway from ^3^(TT) to S_1_ that could alleviate at least some of the losses usually implied by the formation of ^3^(TT).^39^ We therefore use pump–push–probe spectroscopy to investigate the fate of the T_2_ state in rubrene.

### High-Level Reverse Intersystem Crossing

**Figure 7:**
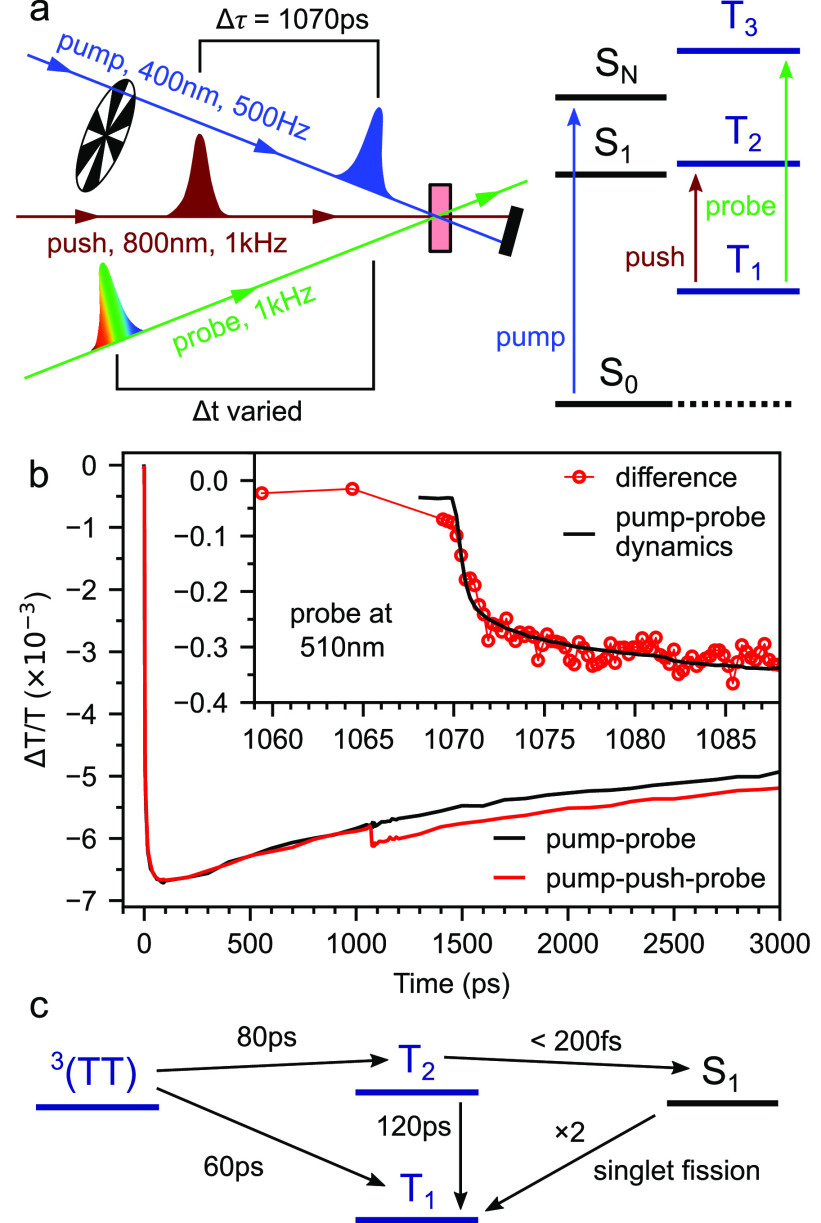
Pump–push–probe spectroscopy of rubrene. (a) Illustration of the pump–push–probe experiment and the electronic transitions targeted by each pulse. The pump initiates singlet fission. After 1 ns, the excited state population will be principally triplets, which are excited from T_1_ to T_2_ by the sub-bandgap push pulse. The probe is used to investigate the effect of the push pulses with and without the initial pump. (b) Pump–probe and pump–push–probe data recorded at a probe wavelength of 510 nm (the T_1_ → T_3_ transition) for a polycrystalline rubrene thin film. The push pulse causes an enhancement of the T_1_ → T_3_ photoinduced absorption with dynamics that match the initial singlet fission. (c) Interpretation of the pump–push–probe data in terms of high-level reverse intersystem crossing from T_2_ to S_1_.

Figure 7a illustrates the pump–push–probe experiment and the transitions in rubrene targeted by each pulse. The 400 nm pump pulses photoexcite the singlet manifold, thereby initiating singlet fission. The push pulses are delayed by a constant 1 ns with respect to the pump, by which time triplets are expected to be the dominant excited states. The sub-bandgap 800 nm push pulses are approximately resonant with the T_1_ → T_2_ transition^76^ and we monitor the probe transmission at 510 nm, which corresponds to T_1_ → T_3_. Other probe wavelengths show no discernible push-induced effects due to reduced signal-to-noise (the triplet PIA is sharply peaked at around 510 nm). These are shown in Supplementary Figure S3. We halve the frequency of the pump pulses only and record the differential transmission as a function of pump–probe delay.

We performed the pump–push–probe experiment on a polycrystalline thin film (characterization in Supplementary Figure S2) rather than the rubrene NPs, because we found it to possess a stronger triplet excited state absorption at 510 nm, giving sufficient signal-to-noise to measure the push-induced effects. Figure 7b shows the results with (red) and without (black) the presence of the push pulses. We find that the push from T_1_ to T_2_ causes an increase, rather than a bleach, of the T_1_ population. Furthermore, the dynamics of the push induced enhancement match the regular pump–probe dynamics of singlet fission, as shown in the inset of Figure 7b.

We consider several possibilities for the underlying photophysics, which we discuss in detail in Supporting Information Section 2.1. First, if the T_2_ states populated by the push pulses simply undergo internal conversion to T_1_, we would expect to see a bleach, and subsequent recovery of the T_1_ population. Alternatively, the push could act as a second pump, perhaps through two-photon absorption.^83,84^ In this case, the ground state population available to be “repumped” by the push is depleted by the first pump pulse, and we would again expect to see a reduction in signal when the push is present. Instead, we observe an enhancement.

We suggest that these results are consistent with recent reports of exothermic high-level reverse intersystem crossing from T_2_ to S_1_ in rubrene.^38^ In this case, T_2_ states populated by the push are converted, via S_1_ and singlet fission, into pairs of triplets. This can only occur in the presence of the initial pump; it therefore manifests itself as an enhancement in the triplet signal rather than a bleach, because each T_2_ state results in a pair of triplets. Quantitative calculations in Supporting Information Section 2.2 further support this assignment. We find that the HL-RISC mechanism should result in a push-induced Δ*T*/*T* signal of between −2.4 × 10^–4^ and −9.3 × 10^–4^. Our measured signal of −3.5 × 10^–4^ falls within this predicted range. We note that the reverse intersystem crossing must occur within the instrument response of our setup (∼200 fs) for the HL-RISC pathway to be consistent with our results. As shown in Figure 7c, we therefore expect HL-RISC to be the dominant fate of the T_2_ excited state because internal conversion to T_1_ is relatively slow owing to the large energy gap (we estimate a time constant of 120 ps from the energy gap law in Figure 6b). Furthermore, we expect singlet fission to be the dominant fate of the resulting S_1_ states, because ISC back to T_2_ is thermally activated and slow (on the order of 1 μs at room temperature^74,85^).

There is precedent for expecting HL-RISC to occur in rubrene. As mentioned above, it is well-known that thermally activated intersystem crossing from S_1_ to T_2_ occurs in rubrene,^74,75^ though estimates of the Arrhenius parameters differ by several orders of magnitude between measurements in solution^74^ and solid glasses.^75^ It must also be possible therefore for the exothermic HL-RISC process to occur. Furthermore, HL-RISC was proposed by several authors to explain high TTA-UC efficiencies in OLED devices based on rubrene^86^ and substituted anthracenes^39,60,87^ (though it is interesting to note that it does not occur in diphenylanthracene (DPA),^39^ perhaps due to symmetry restrictions^88^). Recently, a detailed study of magnetic field effects in rubrene-based OLEDs confirmed that HL-RISC was occurring.^38^ The subpicosecond time scale is also plausible: HL-RISC rate constants for erythrosin B, rose bengal and tetraphenylporphyrin have been measured to be 1 ps or less.^59^ The S_1_–T_2_ energy gaps in these three dyes are several hundred meV greater than in rubrene, so we might expect the HL-RISC rate constant in the latter to be even faster. Finally, we note that vibronic coupling effects have been calculated to increase RISC rate constants by several orders of magnitude in thermally activated delayed fluorescence (TADF) molecules^89^ and suggest that similar effects could help to enable subpicosecond HL-RISC in rubrene.

## Discussion

**Figure 8:**
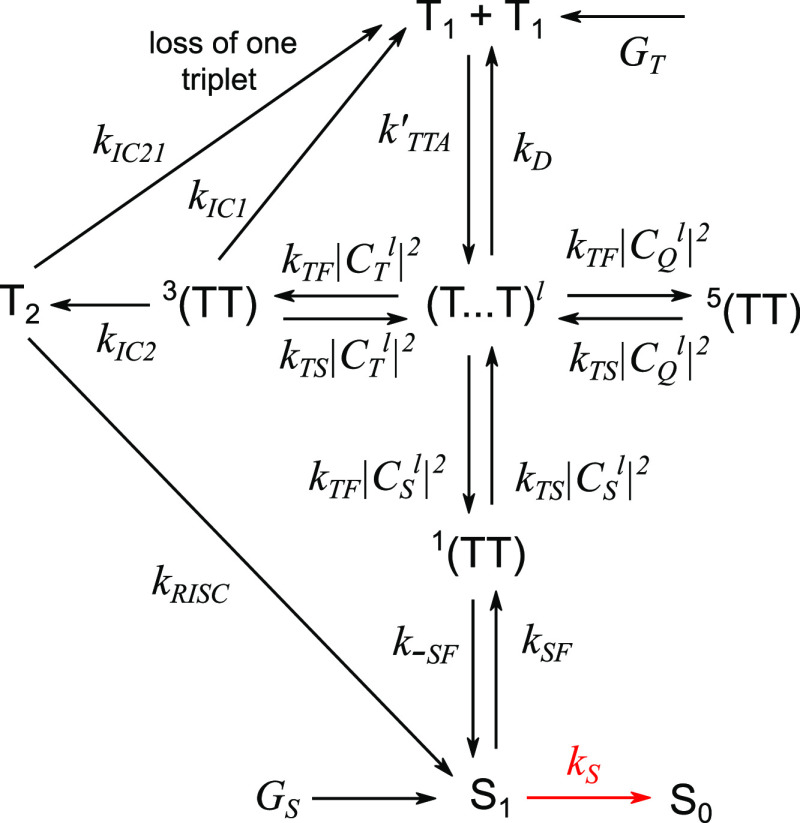
An extended model of TTA-UC. Schematic diagram showing a refinement of the model in Figure 1. We differentiate between triplet-pairs formed directly through TTA, (T···T), and strongly exchange coupled pure spin triplet-pairs, ^1/3/5^(TT). In the limit of strong exchange coupling, these sets of states are identical. We also include a singlet fission channel and provide two distinct internal conversion channels from ^3^(TT). T_2_ can undergo HL-RISC to form S_1_. This is referred to as model 2.

Given that the initial products of TTA are weakly exchange-coupled triplet pairs, and given the important distinction between internal conversions from ^3^(TT) to T_1_ and T_2_, we can extend our simple scheme (model 1) from Figure 1 into that shown in Figure 8 (model 2). Now we explicitly differentiate between triplet-pair states (T···T)^*l*^ formed through TTA, which are governed by the spin Hamiltonian in eq 4, and the pure spin states ^1^(TT), ^3^(TT), and ^5^(TT), which couple to the (T···T)^*l*^ states through their singlet, triplet and quintet character, respectively. In the limit of *J* ≫ *D*, these two sets of states will coincide. We also include a singlet fission channel, and add a distinct T_2_ state that is permitted to undergo HL-RISC to form S_1_.

The rate equations governing this extended model are given in the Supporting Information. The quantum yield of TTA-UC is generally written as^24^
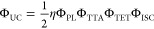
28where Φ_ISC_, Φ_TET_, and Φ_PL_ are the quantum yields of intersystem crossing (or more generally triplet production) on the donor species, triplet energy transfer from donor to acceptor, and acceptor fluorescence, respectively. The factor of  reflects the fact that two triplets yield one singlet and Φ_TTA_ describes the competition between annihilation and decay for the fate of triplet states.

In model 2, by construction Φ_TTA_ = Φ_TET_ = Φ_ISC_ = 1. As a result, the upconversion quantum yield can be calculated from
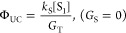
29while the photoluminescence quantum yield is given by
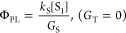
30where *G*_T_ and *G*_S_ are the generation rates for triplet and singlet states, respectively. If the rate constant of singlet fission *k*_SF_ is nonzero, Φ_PL_ may not be unity and instead will depend on the spin statistical factor η, which in general can be calculated as
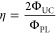
31

Order-of-magnitude values for the main rate constants are given in Supplementary Table S1. As shown Supplementary Figure S4, the values of *k*_TTA_^′^ and *k*_SF_ have no effect on the model predictions, and neither does *G*_*i* = *S or T*_ because the equations are linear. The other rate constants can be varied significantly from the values in Supplementary Table S1 with little impact. Large variations in *k*_TF_, *k*_D_, and *k*_IC_ do have an effect on η, but this is only to be expected^54^ from eq 21. We thus consider the conclusions drawn from the model to be robust and highly general. In our simulations, we use the zero-field splitting parameters of tetracene,^68^*D* = 6.45 × 10^–6^ eV and *E* =–6.45 × 10^–7^ eV and take *X* = *D*/1000.

**Figure 9:**
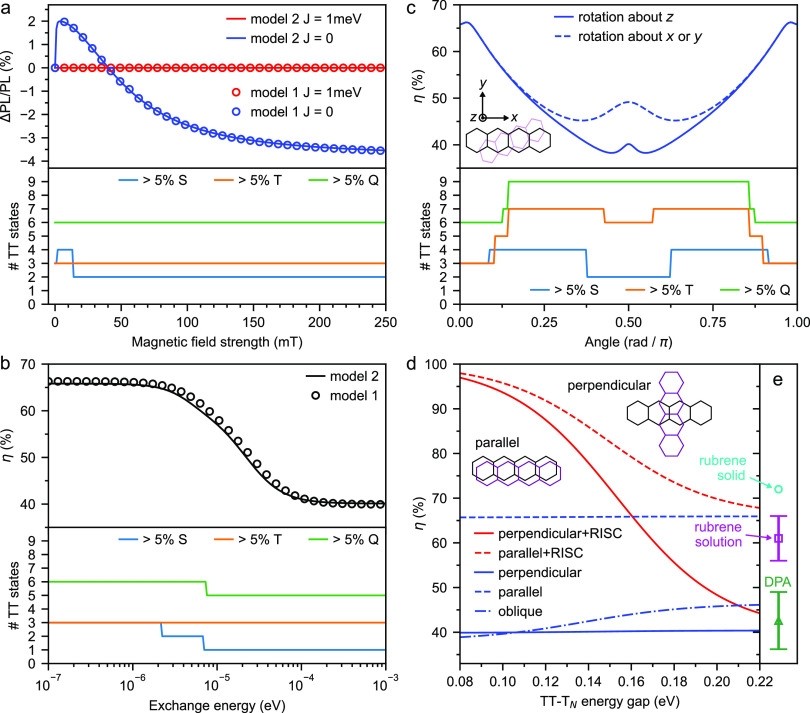
Simulations of factors controlling the spin statistics of TTA. (a) Simulated MFE for parallel molecules comparing model 1 (Figure 1) and model 2 (Figure 8) for strongly (*J* = 1 meV) and weakly (*J* = 0) exchange-coupled triplet-pairs. The lower panel shows changes in spin character of the *J* = 0 triplet-pairs with applied magnetic field. Note that >5% S means triplet-pair states with |*C*_S_^*l*^|^2^ > 5%, i.e. more than 5% singlet character, and similarly for triplet (T) and quintet (Q) character. (b) Simulated spin statistical factor η for parallel molecules as a function of intertriplet exchange energy *J*. The lower panel again shows the changes in triplet-pair spin character. (c) Model 2 simulation showing the variation of η with intermolecular orientation, for *J* = 0 and *k*_RISC_ = 0. The lower panel shows triplet-pair spin character. (d) Model 2 simulation of η as a function of ^3^(TT)-T_N_ energy gap (for rubrene, *N* = 2), for several different cases, all with *J* = 0. The oblique case corresponds to an intermolecular geometry found in the DPA crystal.^90^ (e) Reported experimental ranges of η for DPA, rubrene in solution and rubrene in the solid state, obtained from refs (25, 29, 30, 39, 73, 74, 86, and 91−97). These experimental values, together with reported values of the rubrene T_2_ energy level, are given in Supplementary Tables S2 and S3, respectively.

Figure 9 shows the key predictions from the model. To investigate the effects of intertriplet exchange coupling, we begin by switching off the HL-RISC channel and taking the simplest case of parallel molecules, which corresponds to the π-stacking direction in acene crystals including rubrene. Next, we explore the effects of nonparallel molecular orientation and finally we introduce the HL-RISC channel.

Figure 9a shows the simulated MFE for triplet–triplet annihilation in the limits of strong (red) and weak (blue) exchange coupling. To demonstrate the generality of our model, we also show the (identical) predictions from model 1, incorporating a singlet fission channel (circles). As expected, we find that only in the limit of weak exchange coupling between triplets following TTA do we reproduce the experimentally measured MFE (Figure 4b). The lower panel of Figure 9a illustrates the origin of the *J* = 0 MFE by plotting the number of (T···T)^*l*^ states with |*C*_S_^*l*^|^2^ > 5% (i.e., more than 5% singlet character) as a function of magnetic field, along with equivalent numbers for triplet and quintet character. The threshold of 5% was chosen because it nicely illustrates the key behaviors. At higher fields, two rather than three of the (T···T)^*l*^ triplet-pair states have appreciable singlet character, leading to reduced PL. We note that the HL-RISC channel would introduce further magnetic field effects: the S_1_ states formed can undergo singlet fission, which gives an inverted MFE shape compared to TTA, and the RISC process itself carries a (negative) magnetic field effect^38^ which is beyond the scope of our model.

In Figure 9b, we plot the spin statistical factor for TTA-UC as a function of intertriplet exchange energy. In the conventionally assumed but, as we have explained, incorrect, case of strong exchange coupling, we find the expected limit of . As shown in the lower panel, this is the case for eigenstates that are pure spin states: 5 quintets, 3 triplets, and 1 singlet. The spin statistical factor rises to  as the exchange coupling is reduced, reflecting the increase (from 1 to 3) in the number of triplet-pair states possessing significant singlet character.

As discussed above, the spin character of weakly exchange-coupled triplet-pair states is dependent on the relative orientation of the two molecules involved.^40,43,46^ This has a knock-on effect on the spin statistical factor, as shown in Figure 9c. Rotation of one molecule of the pair with respect to the other causes increased singlet–triplet-quintet mixing. In particular, the greater number of states possessing significant triplet character results in a higher probability for ^3^(TT) → T_N_ internal conversions, thereby reducing the spin statistical factor (in the absence of efficient HL-RISC). The dependence of η on relative molecular orientation may help to explain differences in TTA efficiency between monomeric annihilators and rigid dimers.^98^ Furthermore, it introduces an important consideration for the design of solid state upconversion systems. We find that the parallel orientations associated with close π–π stacking (and hence rapid triplet diffusion^99^) in acene crystals also result in the best spin statistical factors.

Finally, in Figure 9d, we explore the impact of HL-RISC on the spin statistical factor by plotting η against T_2_ energy (relative to the ^3^(TT) level) for several different cases. In solution, the common annihilator molecules rubrene and DPA are thought to form triplet-pair complexes in which the chromophores are oriented perpendicular to each other.^54^ In this case, the spin-statistical factor is 40% in the absence of a HL-RISC channel, but we emphasize that this is a result of weakly interacting triplet-pair states with mixed singlet, triplet and quintet character and not because TTA forms pure singlet, triplet and quintet complexes in a 1:3:5 ratio. We suggest that this is the reason that DPA in solution is reported to give η ∼ 40%.^7,8,39,54,91−95^ The range of experimentally measured values of η for DPA are shown in Figure 9e and the values and references are given in Supplementary Table S2. There are two inequivalent molecules in the DPA crystal unit cell^90^ and therefore two possible triplet-pair orientations, one parallel and one oblique. The oblique orientation results in spin statistical factors within the experimentally reported range.

In rubrene, the HL-RISC channel can contribute due to the favorable energy level alignment between 2 × T_1_, T_2_, and S_1_, which raises the value of η close to the ∼60% measured for rubrene in solution,^25,30^ indicated (with the reported experimental errors) in Figure 9e. In solid rubrene, η has been reported to reach 72%,^86^ also shown in Figure 9e. Again, our model can explain this value through a combination of parallel molecular geometry, weakly exchange-coupled triplet-pairs and a partially active HL-RISC channel. The effectiveness of the HL-RISC channel is highly sensitive to the relative energy levels due to the exponential nature of the energy gap law and Boltzmann factors. Figure 9d shows that variations on the order of *k*_*B*_*T* can have a large impact on η and as shown in Supplementary Table S3, there is a considerable spread in the reported T_2_ energy level of rubrene. Finally, we note that in the absence of HL-RISC, η increases only weakly as T_2_ is raised above ^3^(TT) and never reaches 100% as has been suggested.^28^ In the presence of HL-RISC; however, η = 100% is attained when T_2_ and ^3^(TT) are very close in energy and regardless of intermolecular orientation.

## Conclusions

In this work, we have shown how factors rarely considered in discussions of the spin statistics of TTA can have a profound effect on the efficiency. In particular, we have explained why the oft repeated statement that TTA produces pure singlet, triplet, and quintet encounter complexes in a 1:3:5 ratio contains an implicit assumption that the triplet-pair states are strongly exchange-coupled. This is incompatible with experimentally measured magnetic field effects that can be explained only through weakly exchange-coupled triplet-pair states. When the triplet-pairs are weakly exchange-coupled, our simulations show that varying the intermolecular orientation tunes the spin statistical factor from  for parallel chromophores to  for perpendicular chromophores, through variations in the spin mixing of the triplet-pair wave functions. We suggest that the origin of the commonly observed 40% value for acceptors such as DPA^7,8,39,54,91−95^ is therefore considerably more subtle than has been assumed to date.

Our updated framework for calculating the spin statistical factor can also explain the higher values that have been measured for rubrene. Using transient absorption and pump–push–probe spectroscopy, we provided additional evidence for the recently reported^38^ high-level reverse intersystem crossing channel from T_2_ to S_1_ in rubrene. Based on the energy levels of T_1_, T_2_, and S_1_, we modeled the effect of this channel and found that measured spin statistical factors of 60% for solution^25,30^ and 72% in the solid state^86^ can be readily understood in terms of chromophore orientation and high-level reverse intersystem crossing.

This work points the way toward strategies for exceeding the spin statistical limit of triplet–triplet annihilation. Control of intermolecular distance and geometry within the triplet-pair complexes can result in values up to . Even better, harnessing high-level reverse intersystem crossing can make such considerations redundant, potentially allowing the spin statistical factor to reach unity. These findings therefore provide an important step in understanding that will pave the way for significant efficiency improvements in photon upconverters for solar energy harvesting and light-driven biomedical applications as well as in organic light-emitting diodes.

## Experimental Section

### Preparation of Rubrene Nanoparticles Dispersed in PVA Films

Rubrene, purified by sublimation, was purchased from TCI and used as received. Poly(vinyl alcohol) (PVA, 99+% hydrolyzed, average *M*_*w*_ 130 000) was purchased from Merck and used as received. Films of rubrene nanoparticles (NPs) dispersed in PVA were prepared following previously reported procedures.^65,66^ Briefly, a tetrahydrofuran solution of rubrene (5 mM, 3 mL) was injected into an aqueous solution of sodium dodecyl sulfate (10 mM, 15 mL). The NPs formed were collected by centrifugation and dispersed into an aqueous solution of PVA (8 wt %). The solution was cast onto quartz-coated glass substrates and dried overnight to form films. Prepared films were transferred to a nitrogen-filled glovebox and encapsulated using a glass coverslip and epoxy resin.

### Preparation of Thermally Evaporated Rubrene Films

Rubrene was purchased from Ossila and used as received. Thin films were deposited on precleaned quartz-coated glass substrates by thermal evaporation. The pressure during deposition was 2 × 10^–6^ mbar or lower, the deposition rate was 0.3 Å s^–1^, the source temperature was 174 °C to 177 °C and the final thickness was 125 nm. The fresh, thermally evaporated films appeared smooth and featureless. The films were subsequently annealed on a hot plate at 185 °C for 17 min, resulting in visible crystallization. The polycrystalline films were encapsulated using a glass coverslip and epoxy resin. All preparation was carried out inside a nitrogen-filled glovebox.

### Steady-State Absorption and Time-Resolved Photoluminescence Spectroscopy

Ground state absorption spectra were recorded with a UV–vis spectrophotometer (Cary60, Agilent). A Ti:sapphire regenerative amplifier (Solstice, Spectra-Physics) providing 800 nm pulses (90 fs fwhm, 1 kHz, 4 mJ) was used to generate the pump beam for photoluminescence measurements. A portion of the 800 nm beam was frequency doubled in a BBO crystal to generate 400 nm pump pulses and focused onto the sample. The photoluminescence was detected in reflection geometry by a spectrograph (Shamrock 303i, Andor) and a time-gated intensified charge-coupled device (iCCD; iStar DH334T-18U-73, Andor). A 435 nm long pass filter was used to eliminate pump scatter. Magnetic fields were applied transverse to the excitation beam using an electromagnet. Magnetic field strength was measured using a transverse Hall probe. Data processing procedures and further details regarding the TRPL setup have been reported previously.^37^ The pump beam spot size was measured at the sample position by translating a razor blade through the focus and monitoring the transmitted power.

### Picosecond Transient Absorption Spectroscopy

A Ti:sapphire regenerative amplifier (Spitfire ACE PA-40, Spectra-Physics) providing 800 nm pulses (40 fs full-width at half-maximum (fwhm), 10 kHz, 1.2 mJ) was used to generate both the pump and probe beams. Tunable narrowband pump pulses at 532 nm were generated in an optical parametric amplifier (TOPAS Prime, Light Conversion). The pump was modulated by an optical chopper. Probe pulses spanning the range 350 to 750 nm and 830 to 1200 nm were generated by focusing a portion of the 800 nm beam through a continuously translating calcium fluoride or sapphire crystal, respectively. Pump–probe delay was controlled using a motorized linear stage. Detection was carried out using a commercial instrument (Helios, Ultrafast Systems). The pump and probe polarizations were set to the magic angle. The pump beam spot size was measured at the sample position using a CCD beam profiler (Thorlabs). Transient absorption (TA) spectroscopy data were processed by background subtraction and chirp correction.

### Pump–Push–Probe Spectroscopy

A Ti:sapphire regenerative amplifier (Solstice, Spectra-Physics) providing 800 nm pulses (90 fs fwhm, 1 kHz, 4 mJ) was used to generate the pump, push and probe beams. Probe pulses spanning the range 460 to 700 nm were generated by focusing a portion of the 800 nm beam through a sapphire crystal. A second portion of the 800 nm beam was sent through an optical delay stage, followed by an 80:20 beamsplitter, and used to generate pump and push pulses. The 80% portion was passed through a BBO crystal, short-pass filter (Schott, BG39) and optical chopper to generate pump pulses (400 nm, 500 Hz, 0.2 mJ cm^–2^). The remaining 20% was delayed by a fixed 1070 ps with respect to the pump and used as push pulses (800 nm, 1 kHz, 1.2 mJ cm^–2^). The pump/push and probe polarizations were set to the magic angle and the three beams were overlapped at the sample adjacent to a reference beam obtained by passing the probe through a 50:50 beamsplitter. The reference is used to correct for shot-to-shot variation in the probe spectrum. The probe and reference beams were dispersed by a volume phase holographic grating (Wasastch) and detected by a pair of linear image sensors (S7030, Hamamatsu) driven and read out at the full laser repetition rate by a custom-built board from Entwicklungsbüro Stresing. TA data was acquired using home-built software. The pump and push beam spot sizes were measured at the sample position using a CCD beam profiler (Thorlabs).

## Supporting Information Available

Further analysis of transient absorption data, pump–push–probe spectroscopy of a polycrystalline rubrene film (including discussion of alternative explanations for the pump–push–probe data, why we discount them, and quantitative calculations of the expected pump–push–probe signal), rate equations and constants for the extended kinetic scheme, and literature values of spin statistical factors and energy levels (PDF)
