# Supplementary material for: Spin Statistics for Triplet–Triplet Annihilation Upconversion: Exchange Coupling, Intermolecular Orientation, and Reverse Intersystem Crossing
Source: JACS Au. 2021 Oct 13;1(12):2188–201. doi: 10.1021/jacsau.1c00322 (PMC8715495; doi:10.1021/jacsau.1c00322)
Supplement: Supplementary file 1 — au1c00322_si_001.pdf [file au1c00322_si_001.pdf]

Supplementary Information

Spin statistics for triplet-triplet annihilation upconversion: exchange coupling, intermolecular orientation and reverse intersystem crossing

David G. Bossanyi[1,*], Yoichi Sasaki[2,¶], Shuangqing Wang[1], Dimitri Chekulaev[3], Nobuo Kimizuka[2], Nobuhiro Yanai[2], and Jenny Clark[1,*]

[1]*Department of Physics and Astronomy, The University of Sheffield, Hicks Building, Hounsfield Road, Sheffield, S3 7RH, UK*
[2]*Department of Chemistry and Biochemistry, Graduate School of Engineering, Center for Molecular Systems (CMS), Kyushu University, 744 Moto-oka, Nishi-ku, Fukuoka 819-0395, Japan*
[3]*Department of Chemistry, The University of Sheffield, Dainton Building, Brook Hill, Sheffield, S3 7HF, UK*

[¶]*Present address: University of Strasbourg, CNRS, ISIS, 8 allée G. Monge, 67000, Strasbourg, France*
*E-mail: dgbossanyi1@sheffield.ac.uk, jenny.clark@sheffield.ac.uk

Contents

1 Further analysis of transient absorption data **2**

2 Pump-push-probe spectroscopy of a polycrystalline rubrene film **3**
2.1 Alternative explanations for the pump-push-probe data 3
2.1.1 The push acts as a second pump 4
2.1.2 Internal conversion from T_2 to T_1 4
2.1.3 HL-RISC from T_2 to S_1 4
2.2 Quantitative analysis of the pump-push-probe signal magnitude 5

3 Rate equations and constants for the extended kinetic scheme **7**

4 Literature values of spin statistical factors and energy levels **9**

5 On the fate of strongly exchange-coupled quintet states **10**

Supplementary references **11**

1 Further analysis of transient absorption data

We confirm our assignments (to singlet or triplet states) of spectral features in our transient absorption data by comparing the dynamics.

Fig. S1a shows singlet and triplet PIA spectra in the visible spectral region extracted using multivariate curve resolution alternating least squares (MCR-ALS) [1,2]. Reference spectra used as the starting point of the deconvolution were obtained by time-averaging the data from 0.5–1 ps and 3–7 ns. The concentrations were constrained to be non-negative. The spectra resulting from the MCR-ALS procedure agree well with reported singlet [3] and triplet [4] absorption spectra for rubrene in solution.

The singlet and triplet dynamics extracted using MCR-ALS (Fig. S1b) are characteristic of singlet fission [5]. The triplet population rises with a time constant of approximately 3 ps, accompanied by a 50% reduction in the singlet population with a similar time constant. This suggests that singlet fission ($S_1 \to {}^1(TT)$) and triplet-pair fusion (${}^1(TT) \to S_1$) occur simultaneously with a time constant of $2 \times 3\,\mathrm{ps} = 6\,\mathrm{ps} \sim 10\,\mathrm{ps}$.

In Fig. S1c, we show that the PIA bands at 680 nm and 1170 nm match the singlet dynamics, although an additional fast component is present at 1170 nm. Fig. S1d shows the dynamics at 850 nm and 960 nm, each with the dynamics at 1170 nm, weighted by the absorbance difference between the two wavelengths, subtracted. This removes the singlet component, yielding the residual triplet dynamics, which match those extracted in the visible region by MCR-ALS. This confirms that the peaks at 850 nm and 960 nm arise from triplet excited states.

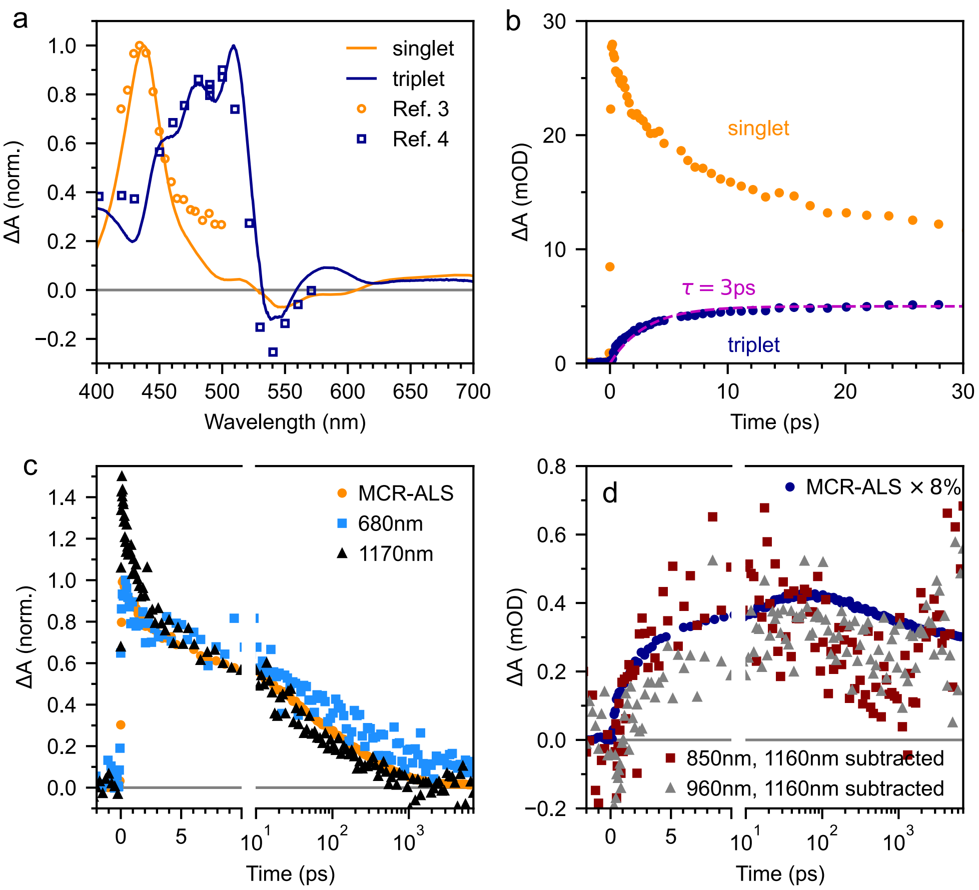

Figure S1. Transient absorption dynamics. (a) Singlet and triplet PIA spectra extracted in the visible region using MCR-ALS [1,2]. The spectra match previously reported singlet [3] and triplet [4] spectra for rubrene in solution. (b) Extracted singlet and triplet dynamics. (c) Comparison of singlet PIA dynamics. (d) Comparison of triplet PIA dynamics. The singlet component has been removed from the dynamics at 850 nm and 960 nm by subtracting the (suitably scaled) dynamics at 1170 nm.

2 Pump-push-probe spectroscopy of a polycrystalline rubrene film

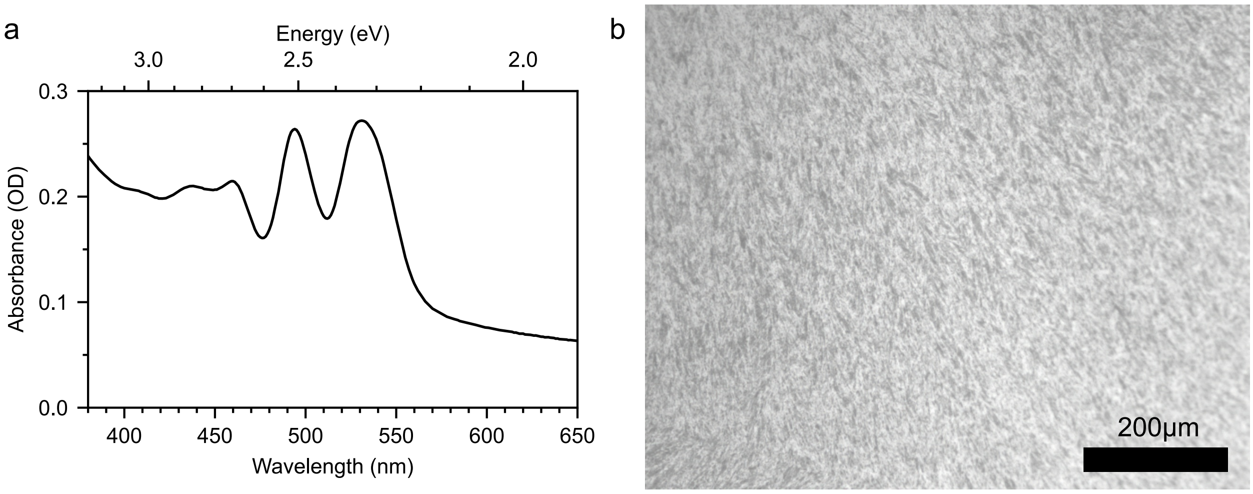

Figure S2. Evaporated polycrystalline rubrene film. (a) Absorption spectrum of the polycrystalline rubrene film. (b) Microscope image of the film surface, showing micron-scale crystalline texture.

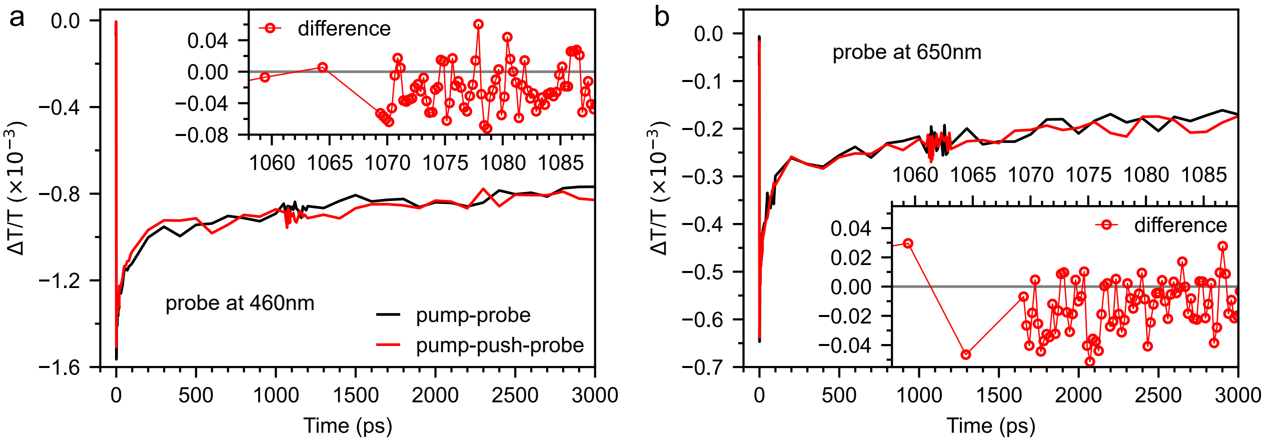

Figure S3. Pump-push-probe spectroscopy at different probe wavelengths. Pump-push-probe spectroscopy for probe wavelengths of 460 nm (a) and 650 nm (b) show no discernable push-induced effects, in contrast to the data at 510 nm (Fig. 7b, main text).

2.1 Alternative explanations for the pump-push-probe data

We find that the effect of our 800 nm push pulses is to enhance the T_1 to T_3 photo-induced absorption (PIA) when the 400 nm pump is present. In this section we investigate the predicted change in triplet PIA for three possible scenarios and show that only the proposed HL-RISC can give rise to an enhancement.

Let the triplet PIA signal X induced by the 400 nm pump have magnitude A at some arbitrary delay time. Then we have

$$X_{0,0} = 0 \quad \text{(S1)}$$

$$X_{1,0} = A, \quad \text{(S2)}$$

where $X_{i,j}$ denotes the signal with the pump on ($i = 1$), pump off ($i = 0$), push on ($j = 1$) or push off ($j = 0$).

Then, for example, the pump-probe signal is given by:

$$X_{pump-probe} = X_{1,0} - X_{0,0} = A - 0 = A. \tag{S3}$$

2.1.1 The push acts as a second pump

The first case we consider is that the push pulse acts as a pump from S_0 to S_N (for example through two-photon absorption) or even from S_0 to T_1. Let the triplet PIA signal induced by the push alone have magnitude B at the same arbitrary delay time:

$$X_{0,1} = B. \tag{S4}$$

When the push is preceded by the 400 nm pump pulse, some of the ground state has already been depleted by the first pump pulse. As a result, the magnitude of the push-induced triplet PIA will be less than B, by an amount δ_1, giving

$$X_{1,1} = X_{1,0} + X_{0,1} - \delta_1 = A + B - \delta_1. \tag{S5}$$

The pump-push-probe signal in this case is

$$X_{pump-push-probe} = X_{1,1} - X_{0,1} = A + B - \delta_1 - B = A - \delta_1, \tag{S6}$$

and so even if the push pulse acts as a second pump, the effect is to reduce the pump-push-probe signal rather than enhance it.

2.1.2 Internal conversion from T_2 to T_1

The second case to consider is that the push pulse excites the T_1 to T_2 transition but that T_2 undergoes internal conversion to T_1. In this case, the push pulse has no effect in the absence of the pump:

$$X_{0,1} = 0, \tag{S7}$$

but causes a reduction δ_2 in the pump-induced PIA due to depletion of the T_1 state:

$$X_{1,1} = X_{1,0} - \delta_2 = A - \delta_2, \tag{S8}$$

and therefore again resulting in a reduced pump-push-probe signal

$$X_{pump-push-probe} = X_{1,1} - X_{0,1} = A - \delta_2 - 0 = A - \delta_2, \tag{S9}$$

where the magnitude δ_2 of the signal reduction would decrease to zero as the T_1 state is repopulated by internal conversion from T_2.

2.1.3 HL-RISC from T_2 to S_1

In this case, the push pulse excites the T_1 to T_2 transition and T_2 undergoes rapid HL-RISC to form S_1. S_1 then undergoes singlet fission, forming T_1+T_1. In the absence of the pump, the push pulse has no effect:

$$X_{0,1} = 0. \tag{S10}$$

When the pump pulse is present, T_1 is again depleted by amount δ_3. However, since each S_1 state formed by the subsequent HL-RISC produces two T_1 states through singlet fission, the triplet PIA is enhanced by an amount $2\delta_3$, giving

$$X_{1,1} = X_{1,0} - \delta_3 + 2\delta_3 = A + \delta_3, \tag{S11}$$

and hence a pump-push-probe signal of

$$X_{pump-push-probe} = X_{1,1} - X_{0,1} = A + \delta_3 - 0 = A + \delta_3. \tag{S12}$$

Thus, HL-RISC is predicted to produce an enhancement of the triplet PIA, and the dynamics of the enhancement should match the pump-probe singlet fission dynamics.

2.2 Quantitative analysis of the pump-push-probe signal magnitude

Having demonstrated that HL-RISC is the most likely explanation for the push-induced enhancement of the triplet PIA, we can estimate the expected magnitude of the enhancement from known and estimated triplet absorption cross sections and measured pulse intensities.

First, we estimate the density of triplet excitons responsible for a given $\Delta T/T$ signal at 510 nm. We can write the triplet PIA absorbance at 510 nm as

$$\Delta A = -\log_{10} e^{-\alpha d} = n_T \sigma d \log_{10}(e), \tag{S13}$$

where α and σ are the triplet absorption coefficient and cross section, respectively, at 510 nm, d is the film thickness and n_T is the number density of triplet excitons.

Transforming into the measurement units of $\Delta T/T$ gives the following expression for the number density of triplet excitons:

$$n_T = \frac{-\log_{10}\left(1 + \frac{\Delta T}{T}\right)}{\sigma d \log_{10}(e)}. \tag{S14}$$

The triplet absorption cross section in the vicinity of 510 nm has been measured as 1.2×10^{-16} cm^2 for rubrene in solution[3]. Using this value, and our film thickness of 125 nm, gives $n_T = 4.5 \times 10^{18}\,\text{cm}^{-3}$ for our maximum $\Delta T/T$ signal of -0.0067 and $n_T = 3.9 \times 10^{18}\,\text{cm}^{-3}$ for $\Delta T/T = -0.0058$, the value just before the arrival of the push pulse (see Fig. 7 in the main text).

We can cross check these triplet exciton densities against the singlet exciton density calculated from the measured pump pulse intensity and the absorption of the film at 400 nm. In general, we have

$$n_S = F_A(1 - F_S)P\frac{\lambda}{hc}\frac{1}{d} \tag{S15}$$

where F_A and F_S are the fraction of incident $\lambda = 400\,\text{nm}$ light absorbed and scattered/reflected by the film, respectively, and P is the pump pulse intensity in units of J cm^{-2}. By fitting a scattering background to the absorption spectrum of the film shown in Fig. S2a, we estimate $F_A \sim 0.1$ and $F_S \sim 0.3$ (from absorbances of 0.05 and 0.15). We measured P to be 0.2 mJ cm^{-2} (see Experimental Section). Using these values in Equation S15 results in an initial photoexcited singlet exciton density of $n_S \sim 2.3 \times 10^{18}\,\text{cm}^{-3}$. Assuming that singlet fission forms triplet excitons with a yield of 200%, we would expect a maximum triplet exciton density of $n_T \sim 4.6 \times 10^{18}\,\text{cm}^{-3}$, which agrees very well with the values calculated above from the solution cross section and the measured $\Delta T/T$ signal.

We are now in a position to estimate the number density of triplet excitons that are re-excited by the push pulse, n'_T, and hence obtain estimates for the push-induced $\Delta T/T$ signal from Equation S14.

The number density of triplets re-excited by the push can be evaluated from Equation S15, where P now represents the $\lambda = 800\,\text{nm}$ push pulse intensity which we measured to be 1.2 mJ cm^{-2}. F_S is now the fraction of 800 nm light that is scattered by the film which we again obtain from the absorption spectrum, finding $F_S \sim 0.09$. The unknown parameter is F_A, the fraction of 800 nm light absorbed by the $T_1 \rightarrow T_2$ transition in the film. We have

$$F_A = 1 - 10^{-A_{800}}, \tag{S16}$$

where the triplet-triplet absorbance at 800 nm is given by:

$$A_{800} = n_T \sigma' d \log_{10}(e) = n_T \sigma \frac{\sigma'}{\sigma} d \log_{10}(e). \quad \text{(S17)}$$

Here σ and σ' are the triplet absorption cross sections at 510 nm and 800 nm respectively and $n_T = 3.9 \times 10^{18}\,\mathrm{cm}^{-3}$ (see above) is the triplet exciton density just before the arrival of the push pulse.

The ratio of triplet-triplet absorption cross sections at 510 nm and 800 nm can be estimated from measured triplet PIA spectra for rubrene. We find significant variation between different measurements. For example the triplet PIA spectrum reported by Miyata et al.[6] for rubrene single crystals gives $\sigma'/\sigma \sim 0.3$. We suggest that this is an upper bound, given the significant spectral overlap between singlet and triplet PIA bands in the vicinity of 800 nm in their data. We can obtain a lower bound from our own transient absorption data in Fig. 5, main text. Although we cannot use the data at 800 nm due to residual fundamental in that probe region, we can take the ratio of the PIA signal at 510 nm and 850 nm (for $t > 1000\,\mathrm{ps}$) as a lower bound, giving $\sigma'/\sigma \sim 0.08$.

These upper and lower bounds for the triplet-triplet cross section ratio result in a range of $1.6 \times 10^{17}\,\mathrm{cm}^{-3} < n'_T < 6.2 \times 10^{17}\,\mathrm{cm}^{-3}$ for the push-induced triplet density, in other words the T_2 exciton density caused by the push pulse. In the HL-RISC picture, pushing from T_1 to T_2 results in the net gain of one T_1. This is because the push removes one T_1 state but HL-RISC from T_2 to S_1, followed by singlet fission from S_1 to T_1+T_1, adds back two T_1 states.

Thus we can simply convert n'_T into an expected push-induced $\Delta T/T$ signal at 510 nm by rearranging Equation S14. Assuming that all T_2 states populated by the push undergo HL-RISC, we predict that the magnitude of the push-induced $\Delta T/T$ signal should lie between -2.4×10^{-4} and -9.3×10^{-4}. Our measured value of -3.5×10^{-4} (see Fig. 7b, main text) lies within this range, providing additional justification for our interpretation of the pump-push-probe data in terms of HL-RISC.

3 Rate equations and constants for the extended kinetic scheme

The rate equations for model 2 (Fig. 8 in the main text) can be written as follows:

$$\frac{d[\mathrm{T}_1]}{dt} = G_T + 2k_D \sum_{l=1}^{9}[(\mathrm{T}...\mathrm{T})^l] + k_{IC1} \sum_{m=x,y,z} |C_T^l|^2[^3(\mathrm{TT})_m] + k_{IC21}[\mathrm{T}_2] - 2k'_{TTA}[\mathrm{T}_1] \tag{S18}$$

$$\begin{aligned}\frac{d[(\mathrm{T}...\mathrm{T})^l]}{dt} &= \frac{1}{9}k'_{TTA}[\mathrm{T}_1] - k_D[(\mathrm{T}...\mathrm{T})^l] - k_{TF}\left(|C_S^l|^2 + |C_T^l|^2 + |C_Q^l|^2\right)[(\mathrm{T}...\mathrm{T})^l] \\ &+ k_{TS}\left(|C_S^l|^2[^1(\mathrm{TT})] + \sum_{m=x,y,z}|C_{Tm}^l|^2[^3(\mathrm{TT})_m] + \sum_{m=a,b,x,y,z}|C_{Qm}^l|^2[^5(\mathrm{TT})_m]\right)\end{aligned} \tag{S19}$$

$$\frac{d[^5(\mathrm{TT})_m]}{dt} = k_{TF}\sum_{l=1}^{9}|C_{Qm}^l|^2[(\mathrm{T}...\mathrm{T})^l] - k_{TS}\left(\sum_{l=1}^{9}|C_{Qm}^l|^2\right)[^5(\mathrm{TT})_m] \tag{S20}$$

$$\frac{d[^3(\mathrm{TT})_m]}{dt} = k_{TF}\sum_{l=1}^{9}|C_{Tm}^l|^2[(\mathrm{T}...\mathrm{T})^l] - k_{TS}\left(\sum_{l=1}^{9}|C_{Tm}^l|^2\right)[^3(\mathrm{TT})_m] - (k_{IC1} + k_{IC2})[^3(\mathrm{TT})_m] \tag{S21}$$

$$\frac{d[^1(\mathrm{TT})]}{dt} = k_{TF}\sum_{l=1}^{9}|C_S^l|^2[(\mathrm{T}...\mathrm{T})^l] - k_{TS}\left(\sum_{l=1}^{9}|C_S^l|^2\right)[^1(\mathrm{TT})] + k_{SF}[\mathrm{S}_1] - k_{-SF}[^1(\mathrm{TT})] \tag{S22}$$

$$\frac{d[\mathrm{S}_1]}{dt} = G_S + k_{-SF}[^1(\mathrm{TT})] - k_{SF}[\mathrm{S}_1] - k_S[\mathrm{S}_1] + k_{RISC}[\mathrm{T}_2] \tag{S23}$$

$$\frac{d[\mathrm{T}_2]}{dt} = k_{IC2}\sum_{m=x,y,z}|C_T^l|^2[^3(\mathrm{TT})_m] - (k_{IC21} + k_{RISC})[\mathrm{T}_2]. \tag{S24}$$

Since we assumed an effective linear annihilation rate constant k'_{TTA}, the equations were solved numerically using linear algebra. Definitions of rate constants and overlap factors are given in the main text. Table S1 gives the values of the rate constants used in the simulations and indicates the source of each one. Fig. S4 shows the sensitivity of the model to different rate constants.

Table S1. Values and sources of the main rate constants.

Rate	Value (ns^{-1})	Source
k_S	0.0625	Refs. 7,8
k_{SF}	100	Approximated from TA data (Fig. S1)
k_{-SF}	100	Approximated from TA data (Fig. S1)
k_{TS}	10	Lower bound on triplet hopping rate constant, estimated from the TTA rate constant in Ref. 9, larger values have no effect (Fig. S4)
k_{TF}	5	$= k_{TS}/2$ for 1D diffusion
k_D	0.1	Approximate, estimated from onset of bimolecular TTA in Fig. 4
k_{IC1}	17	Energy gap law (Fig. 6)
k_{IC2}	12	Energy gap law (Fig. 6)
k_{IC21}	8	Energy gap law (Fig. 6)
k_{RISC}	5000	Approximate instrument response of pump-push-probe

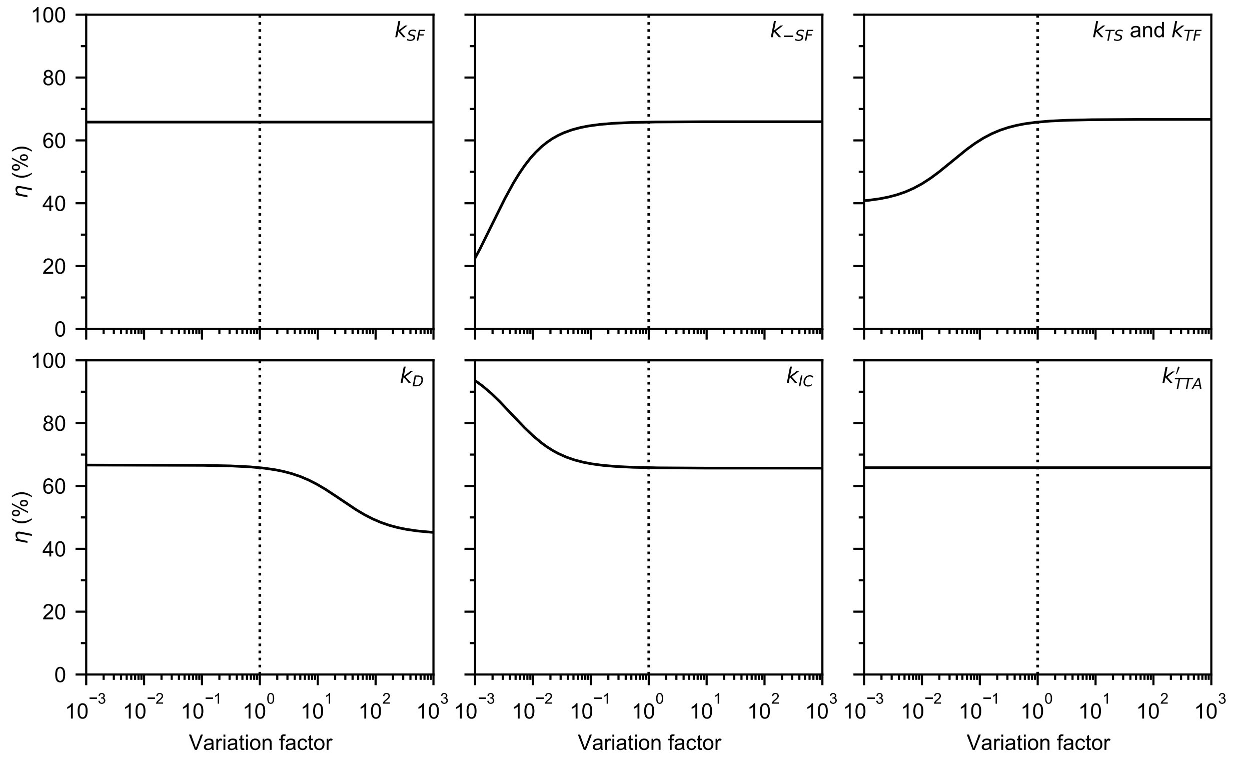

Figure S4. Sensitivity of model 2 to different rate constants. Each rate constant was varied by ± 3 orders of magnitude about its original value and the effect on η monitored. In particular, we highlight that the value of η does not depend on k_{SF} or k'_{TTA} and that increasing the triplet hopping rate constants further also has little impact. Note that k_{IC} is the sum of K_{IC1} and k_{IC2}.

4 Literature values of spin statistical factors and energy levels

Supplementary Tables S2 and S3 give non-exhaustive experimental literature values for the spin statistical factor of diphenylanthracene (DPA) and rubrene and the T_2 energy in rubrene. These values were used to indicate the spread of reported experimental values shown on Fig. 9d of the main text.

Table S2. Experimental values of the spin statistical factor for rubrene and DPA.

Annihilator	System	η (%)	Reference
DPA	thin film	37	10
DPA	solution	44^a	11
DPA	solution	36^a	12
DPA	solution	45	13
DPA	solution	36^a	14
DPA	solution	48^a	15
DPA	solution	52	16
rubrene	solid	72	17
rubrene	solution	$61(5)^b$	18,19
rubrene	solution	15^c	20

^{a}The value given is the upconversion quantum yield, which is very close to η since the other efficiencies are near unity.
^{b}The value and error are taken from the measurements in Ref. 18. Very similar values were reported in Ref. 19
^{c}It is unclear why the values reported in Ref. 20 are so much lower than the others.

Table S3. Experimental values of the T_2 energy level in rubrene. Note that only near-infrared (NIR) transient absorption (TA) data is included: some authors report the $T_1 \rightarrow T_2$ transition at 800 nm[6,21], giving a T_2 energy of 2.69 eV however their probe spectra do not extend far enough into the NIR to determine the true transition energy. ISC stands for intersystem crossing.

System	Method	T_2 energy (eV)	Reference
solution	thermally activated ISC	2.38-2.40	22
thin film	NIR TA	2.40	23
nanoparticle film	NIR TA	2.43	This work
crystal	NIR TA	2.55	24

5 On the fate of strongly exchange-coupled quintet states

Strongly exchange-coupled quintet triplet-pair states in singlet fission systems, i.e. ^{5}(TT), were first directly observed by time-resolved electron paramagnetic resonance (trEPR) in 2016, both for TIPS-tetracene thin films[25] and pentacene-bridge-pentacene dimers[26]. Singlet and quintet triplet-pair states are mixed under weak exchange coupling. As such, various mechanisms have been proposed for the formation of ^{5}(TT) from ^{1}(TT), all based on fluctuating inter-triplet exchange interactions[25,27]. For instance, the hopping of one triplet in the pair onto a neighbouring molecule necessarily weakens the exchange coupling[28–30]. Alternative suggestions include polaronic (exciton-lattice) distortion, molecular relaxation or hopping to sites with different J[25]. The latter mechanism has been used to explain the changes in fluorescence of DPH crystals under strong magnetic fields[31] and is supported by the identification of several distinct triplet-pair sites in TIPS-tetracene with differing exchange interactions[30,32].

Several recent trEPR studies have claimed that ^{1}(TT) and ^{5}(TT) can interconvert[33–36]. However, we agree with Atkins and Evans[37] that a direct interconversion is suppressed under strong exchange coupling and that instead the conversion from ^{1}(TT) to ^{5}(TT) and vice versa is mediated by weakly exchange-coupled singlet-quintet mixtures, i.e. $^{1/5}$(T...T). This kind of mediated interconversion is explicitly included in model 2 (Fig. 8, main text) in which we clearly distinguish between weakly and strongly exchange coupled triplet-pair states.

EPR experiments are sensitive only to pure, non-zero spin states. As such, whilst $^{1/5}$(T...T) states do not show up in these experiments that does not mean that they do not play a role in the dynamics.

Supplementary references

[1] Jaumot, J., Gargallo, R., de Juan, A. & Tauler, R. A graphical user-friendly interface for MCR-ALS: a new tool for multivariate curve resolution in MATLAB. *Chemom. Intell. Lab. Syst.* **76**, 101–110 (2005).

[2] Jaumot, J., de Juan, A. & Tauler, R. MCR-ALS GUI 2.0: New features and applications. *Chemom. Intell. Lab. Syst.* **140**, 1–12 (2015).

[3] Löhmannsröben, H. G. Photophysical properties and laser performance of rubrene. *Appl. Phys. B* **47**, 195–199 (1988).

[4] Yee, W. A., Kuzmin, V. A., Kliger, D. S., Hammond, G. S. & Twarowski, A. J. Quenching of the fluorescent state of rubrene directly to the ground state. *J. Am. Chem. Soc.* **101**, 5104–5106 (1979).

[5] Ma, L. *et al.* Singlet fission in rubrene single crystal: direct observation by femtosecond pump–probe spectroscopy. *Phys. Chem. Chem. Phys.* **14**, 8307–8312 (2012).

[6] Miyata, K. *et al.* Coherent singlet fission activated by symmetry breaking. *Nat. Chem.* **9**, 983–989 (2017).

[7] Strickler, S. J. & Berg, R. A. Relationship between absorption intensity and fluorescence lifetime of molecules. *J. Chem. Phys.* **37**, 814–822 (1962).

[8] Finton, D. M., Wolf, E. A., Zoutenbier, V. S., Ward, K. A. & Biaggio, I. Routes to singlet exciton fission in rubrene crystals and amorphous films. *AIP Adv.* **9**, 95027 (2019).

[9] Biaggio, I. & Irkhin, P. Extremely efficient exciton fission and fusion and its dominant contribution to the photoluminescence yield in rubrene single crystals. *Appl. Phys. Lett.* **103**, 263301 (2013).

[10] Ieuji, R., Goushi, K. & Adachi, C. Triplet–triplet upconversion enhanced by spin–orbit coupling in organic light-emitting diodes. *Nat. Commun.* **10**, 5283 (2019).

[11] Monguzzi, A. *et al.* High efficiency up-converting single phase elastomers for photon managing applications. *Adv. Energy Mater.* **3**, 680–686 (2013).

[12] Khnayzer, R. S. *et al.* Upconversion-powered photoelectrochemistry. *Chem. Commun.* **48**, 209–211 (2012).

[13] MacQueen, R. W., Cheng, Y. Y., Danos, A. N., Lips, K. & Schmidt, T. W. Action spectrum experiment for the measurement of incoherent photon upconversion efficiency under sun-like excitation. *RSC Adv.* **4**, 52749–52756 (2014).

[14] Yanai, N. *et al.* Absolute method to certify quantum yields of photon upconversion via triplet–triplet annihilation. *J. Phys. Chem. A* **123**, 10197–10203 (2019).

[15] Olesund, A., Gray, V., Mårtensson, J. & Albinsson, B. Diphenylanthracene dimers for triplet–triplet annihilation photon upconversion: Mechanistic insights for intramolecular pathways and the importance of molecular geometry. *J. Am. Chem. Soc.* **143**, 5745–5754 (2021).

[16] Monguzzi, A., Tubino, R., Hoseinkhani, S., Campione, M. & Meinardi, F. Low power, non-coherent sensitized photon up-conversion: modelling and perspectives. *Phys. Chem. Chem. Phys.* **14**, 4322 (2012).

[17] Kondakov, D. Y., Pawlik, T. D., Hatwar, T. K. & Spindler, J. P. Triplet annihilation exceeding spin statistical limit in highly efficient fluorescent organic light-emitting diodes. *J. Appl. Phys.* **106**, 124510 (2009).

[18] Cheng, Y. Y. *et al.* Kinetic analysis of photochemical upconversion by triplet-triplet annihilation: Beyond any spin statistical limit. *J. Phys. Chem. Lett.* **1**, 1795–1799 (2010).

[19] Cheng, Y. Y. *et al.* On the efficiency limit of triplet–triplet annihilation for photochemical upconversion. *Phys. Chem. Chem. Phys.* **12**, 66–71 (2010).

[20] Radiunas, E. *et al.* Understanding the limitations of NIR-to-visible photon upconversion in phthalocyanine-sensitized rubrene systems. *J. Mater. Chem. C* **8**, 5525–5534 (2020).

[21] Zhu, T., Wan, Y., Guo, Z., Johnson, J. & Huang, L. Two birds with one stone: Tailoring singlet fission for both triplet yield and exciton diffusion length. *Adv. Mater.* **28**, 7539–7547 (2016).

[22] Lewitzka, F. & Löhmannsröben, H.-G. Investigation of triplet tetracene and triplet rubrene in solution. *Zeitschrift für Phys. Chemie* **150**, 69–86 (1986).

[23] Yong, C. K. *et al.* The entangled triplet pair state in acene and heteroacene materials. *Nat. Commun.* **8**, 15953 (2017).

[24] Bera, K., Douglas, C. J. & Frontiera, R. R. Femtosecond Raman microscopy reveals structural dynamics leading to triplet separation in rubrene singlet fission. *J. Phys. Chem. Lett.* **8**, 5929–5934 (2017).

[25] Weiss, L. R. *et al.* Strongly exchange-coupled triplet pairs in an organic semiconductor. *Nat. Phys.* **13**, 176–181 (2017).

[26] Tayebjee, M. J. Y. *et al.* Quintet multiexciton dynamics in singlet fission. *Nat. Phys.* **13**, 182–188 (2017).

[27] Collins, M. I., McCamey, D. R. & Tayebjee, M. J. Y. Fluctuating exchange interactions enable quintet multiexciton formation in singlet fission. *J. Chem. Phys.* **151**, 164104 (2019).

[28] Pensack, R. D. *et al.* Observation of two triplet-pair intermediates in singlet exciton fission. *J. Phys. Chem. Lett.* **7**, 2370–2375 (2016).

[29] Scholes, G. D. Correlated pair states formed by singlet fission and exciton–exciton annihilation. *J. Phys. Chem. A* **119**, 12699–12705 (2015).

[30] Taffet, E. J., Beljonne, D. & Scholes, G. D. Overlap-driven splitting of triplet pairs in singlet fission. *J. Am. Chem. Soc.* **142**, 20040–20047 (2020).

[31] Yago, T., Ishikawa, K., Katoh, R. & Wakasa, M. Magnetic field effects on triplet pair generated by singlet fission in an organic crystal: Application of radical pair model to triplet pair. *J. Phys. Chem. C* **120**, 27858–27870 (2016).

[32] Bayliss, S. L. *et al.* Site-selective measurement of coupled spin pairs in an organic semiconductor. *Proc. Natl. Acad. Sci.* **115**, 5077–5082 (2018).

[33] Basel, B. S. *et al.* Unified model for singlet fission within a non-conjugated covalent pentacene dimer. *Nat. Commun.* **8**, 15171 (2017).

[34] Lubert-Perquel, D. *et al.* Identifying triplet pathways in dilute pentacene films. *Nat. Commun.* **9**, 4222 (2018).

[35] Chen, M. *et al.* Quintet-triplet mixing determines the fate of the multiexciton state produced by singlet fission in a terrylenediimide dimer at room temperature. *Proc. Natl. Acad. Sci.* **116**, 8178–8183 (2019).

[36] Bae, Y. J. *et al.* Spin dynamics of quintet and triplet states resulting from singlet fission in oriented terrylenediimide and quaterrylenediimide films. *J. Phys. Chem. C* **124**, 9822–9833 (2020).

[37] Atkins, P. & Evans, G. Magnetic field effects on chemiluminescent fluid solutions. *Mol. Phys.* **29**, 921–935 (1975).
